# Dietary Patterns Differently Associate with Inflammation and Gut Microbiota in Overweight and Obese Subjects

**DOI:** 10.1371/journal.pone.0109434

**Published:** 2014-10-20

**Authors:** Ling Chun Kong, Bridget A. Holmes, Aurelie Cotillard, Fatiha Habi-Rachedi, Rémi Brazeilles, Sophie Gougis, Nicolas Gausserès, Patrice D. Cani, Soraya Fellahi, Jean-Philippe Bastard, Sean P. Kennedy, Joel Doré, Stanislav Dusko Ehrlich, Jean-Daniel Zucker, Salwa W. Rizkalla, Karine Clément

**Affiliations:** 1 INSERM, UMR_S U1166, Nutriomics, Paris, France; Sorbonne Universités, Université Pierre et Marie Curie-Paris, Paris, France; 2 Institute of Cardiometabolism and Nutrition (ICAN), Assistance Publique-Hôpitaux de Paris, Heart and Nutrition Department, and Human Nutrition Research Center-Ile de France, Hôpital Pitié-Salpêtrière, Paris, France; 3 Danone Research, RD 128, Palaiseau, France; 4 IT&M STATS, Paris, France; 5 Université catholique de Louvain, Louvain Drug Research Institute, WELBIO (Walloon Excellence in Life sciences and BIOTechnology), Metabolism and Nutrition Research group, Brussels, Belgium; 6 Assistance Publique Hôpitaux de Paris, Service de Biochimie et Hormonologie, Hôpital Tenon, Paris, France; 7 Institut National de la Recherche Agronomique, UMR 1319 MICALIS, Jouy en Josas, France; University College Dublin, Ireland

## Abstract

**Background:**

Associations between dietary patterns, metabolic and inflammatory markers and gut microbiota are yet to be elucidated.

**Objectives:**

We aimed to characterize dietary patterns in overweight and obese subjects and evaluate the different dietary patterns in relation to metabolic and inflammatory variables as well as gut microbiota.

**Design:**

Dietary patterns, plasma and adipose tissue markers, and gut microbiota were evaluated in a group of 45 overweight and obese subjects (6 men and 39 women). A group of 14 lean subjects were also evaluated as a reference group.

**Results:**

Three clusters of dietary patterns were identified in overweight/obese subjects. Cluster 1 had the least healthy eating behavior (highest consumption of potatoes, confectionary and sugary drinks, and the lowest consumption of fruits that was associated also with low consumption of yogurt, and water). This dietary pattern was associated with the highest LDL cholesterol, plasma soluble CD14 (p = 0.01) a marker of systemic inflammation but the lowest accumulation of CD163+ macrophages with anti-inflammatory profile in adipose tissue (p = 0.05). Cluster 3 had the healthiest eating behavior (lower consumption of confectionary and sugary drinks, and highest consumption of fruits but also yogurts and soups). Subjects in this Cluster had the lowest inflammatory markers (sCD14) and the highest anti-inflammatory adipose tissue CD163+ macrophages. Dietary intakes, insulin sensitivity and some inflammatory markers (plasma IL6) in Cluster 3 were close to those of lean subjects. Cluster 2 was in-between clusters 1 and 3 in terms of healthfulness. The 7 gut microbiota groups measured by qPCR were similar across the clusters. However, the healthiest dietary cluster had the highest microbial gene richness, as evaluated by quantitative metagenomics.

**Conclusion:**

A healthier dietary pattern was associated with lower inflammatory markers as well as greater gut microbiota richness in overweight and obese subjects.

**Trial Registration:**

ClinicalTrials.gov NCT01314690

## Introduction

Dietary pattern analysis is a useful way to consider the diet as a whole, rather than considering individual foods or nutrients. The analysis of dietary patterns provides an opportunity to investigate relationships between diet and health in nutritional epidemiology [1]–[3]. Some prospective studies have demonstrated a relationship between dietary patterns and weight changes [4]–[6], although not all studies are consistent [7].

Dietary patterns have also been associated with markers of systemic inflammation and risk of cardiovascular diseases [8]–[10]. A healthy dietary pattern (higher in fruits, vegetables, poultry, tea, fruit juices and whole grains) was found inversely related to systemic C-reactive protein (CRP), while a western dietary pattern was positively related to CRP in different populations [8]–[11]. A similar healthy eating pattern was also associated with reduced insulin resistance [12] and the risk of metabolic syndrome [10], [13]. The mechanisms behind these links still have to be elucidated. The gut microbiota is viewed as a pivotal actor linking “external” macro-environmental changes to the “internal” host biology particularly inflammation as well as metabolic and body weight homeostasis. Gut microbiota has been shown to be involved in the development of metabolic syndrome and low-grade inflammation associated with obesity via different mechanisms including lipopolysaccarides-Toll-like receptors/CD14 (LPS-TLRs/CD14) complex mostly in animal models [14]. To our knowledge, a limited number of studies have investigated the relationship between dietary patterns, gut microbiota, and host inflammatory levels in humans.

Recently, quantitative metagenomic approaches have provided the opportunity to study the gut microbiota in humans in more depth. Interestingly, a rapid adaptation of gut microbiota could be observed after 24 h of switching from a low-fat, plant polysaccharide-rich diet to a high fat high sugar “western” diet [15], whereas the diversity of species within the gut microbiota organized into identifiable clusters or enterotypes are correlated with long-term but not short term dietary patterns [16]. We found recently that when subjects were clustered in function of their bacterial gene count, low fecal bacterial gene richness was associated with impaired glucose homeostasis and higher low-grade inflammation during a weight loss dietary program in overweight/obese subjects [17]. Low gene richness might not only be linked with isolated foods, but rather with global dietary patterns, an aspect not yet explored in the later study. Therefore we aimed to explore in the same cohort of subjects at baseline before any dietary transition, the relationship between different dietary patterns, metabolic and inflammatory variables and gut microbiota evaluated by qPCR and next-generation sequencing methods. The results in the overweight/obese group were compared to a group of lean subjects.

## Materials and Methods

The protocol for this trial and supporting CONSORT checklist are available as supporting information; see Checklist S1 and Protocol S1.

### Subjects

**Figure 1:**
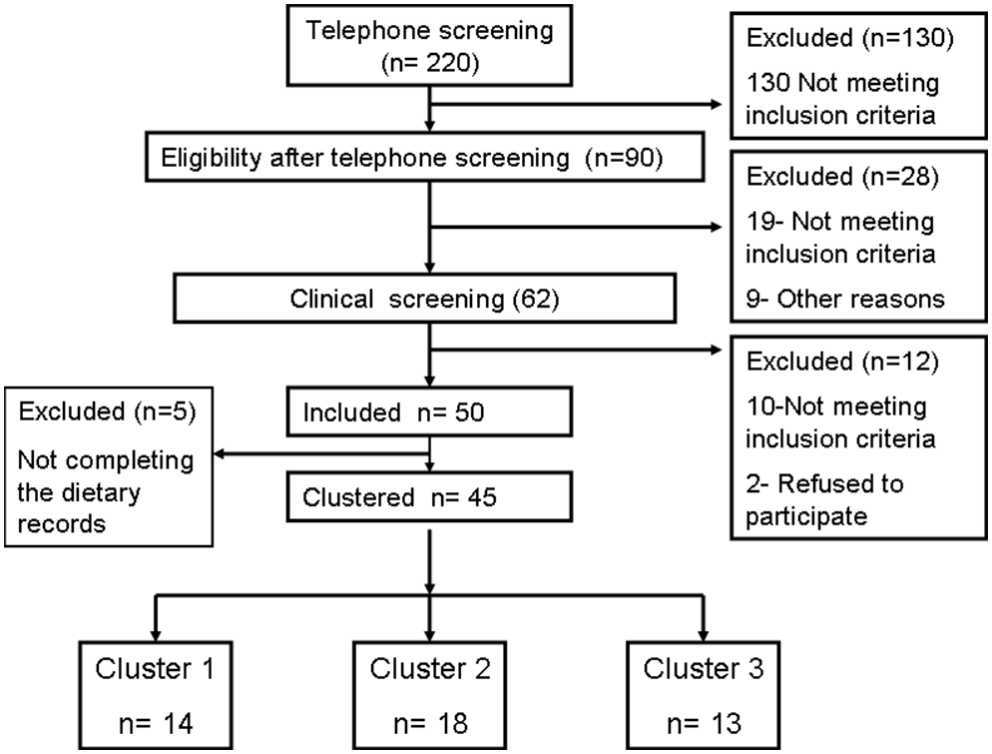
Consort Flowchart.

Fifty overweight and obese (BMI: ≥25−<38 Kg/m^2^), but otherwise healthy subjects (8 males and 42 females), aged from 25 to 65 years, were recruited at the Human Nutrition Research Center (Pitié-Salpêtrière Hospital, Paris, France). Forty-five subjects (6 males and 39 females) completed the 7-day dietary records, bioclinical and fecal bacterial data and were included in the present analysis (Figure 1). Selection of subjects was based on the absence of inflammatory or infectious diseases, cancer or any history of gastrointestinal problems. Subjects with hepatic, renal or cardiac diseases were excluded. In addition, 17 normal weight healthy female (BMI: <25 >18 kg/m^2^) volunteers living in the same area as the obese subjects were recruited as a control group. Fourteen of these completed the dietary records and were included for reference purposes. No antibiotics were taken within 2-months prior to the start of the study. Subjects were enrolled between October 2008 and December 2009. The Ethical Committee of Hotel-Dieu Hospital, Paris, France, approved the protocol. All the subjects gave written informed consent. This clinical trial was registered before enrolment of participants in the EU Clinical trials Register under the identification number: 2008-001138-28, and in the French agency for the security of medications and health products - changed to ANSM) under the identification number: ID RCB 2008-A00406-49. It was also registered in the ClinicalTrials.gov under the identification number: NCT01314690, later due to some technical problems with the sponsor of the study. The authors confirm that all ongoing and related trials for this study are registered.

All selected participants underwent a series of tests following an overnight fast. Blood samples were taken to measure plasma glucose, insulin, lipids and some inflammatory markers. Body fat and fat-free mass distributions were measured using dual-energy X-ray absorptiometry (Hologic APEX, discovery W. (S/N 84030), version 3.0, Bedford, MA). Estimation of pancreatic β-cell function (HOMA B%) and insulin sensitivity (HOMA S%) were calculated using homeostasis model assessment: HOMA/CIGMA software.

### Dietary assessment

Dietary intake was assessed using an unweighed dietary record for 7-days (unless specified otherwise). The records were self-completed and contained pre-defined spaces for the recording of each meal. Subjects were provided with instructions on how to complete the dietary records. On the visit day, the dietitian verified the information with the subject. All records were analyzed by using the computer software program PROFILE DOSSIER X029 (Audit Conseil en Informatique Médicale, Bourges, France), which has a food composition database initially made up of 400 food items representative of the French diet as described previously [18]. Nutrient intakes were generated for each subject based on the dietary data collected. Information on consumed foods could not be directly extrapolated from the program and were therefore coded manually into 26 food groups. Food groups were defined based on groups and sub-groups typically used in the reporting of dietary data in large surveys, such as the French Nutrition and Health Survey and program (Étude Nationale Nutrition Santé, ENNS) [19], [20]. Mean food and nutrient intakes in the overweight/obese subjects were calculated for each subject according to the 7-day diary (in 2 subjects only 6 days of data were available). The lean subjects completed a 3-day diary of the same format as those used for overweight and obese subjects. Habitual physical activity (Baecke questionnaire) was also evaluated.

### Adipocyte morphology and immunohistochemical analysis

Subcutaneous abdominal adipose tissue samples were obtained by needle biopsy from the periumbilical area under local anesthesia (1% xylocaine) for measuring adipocyte diameter and immunohistochemical detection (HAM56, CD163). Sub-cutaneous adipose tissue samples were washed in physiological saline. A fresh aliquot was used to measure adipocyte diameter as described previously [21] by using Perfect Image software (Claravision. Orsay.France). Other aliquots were fixed overnight at 4°C in 4% paraformaldehyde, and then embedded in paraffin for immune histochemical detection. Antibody anti-HAM56 and anti-CD163 (DakoCytomation, Trappes, France) were used to target the macrophage cells in subcutaneous adipose tissue by the avidin-biotin-peroxidase method. The number of positively marked cells was counted by two blinded investigators as previously described [22]. We calculated the labeled cell number per field, and the adjusted number (percentage per 100 adipocytes).

### Gut Microbiota

(qPCR and SOLiD methods).

Fecal samples were obtained on the morning of the study before breakfast after 12 hours of fasting. Whole fecal samples were self-collected in sterile boxes and stored at −20°C within 4 h. Samples were treated in the laboratory as 200-mg aliquots and stored at −80°C until further analysis. DNA were extracted using the method previously described [23].

#### Real time qPCR method

The procedure for qPCR was performed as previously described (23). Seven bacterial groups were detected: *Clostridium leptum* (*C. leptum*), *Clostridium coccoides* (*C. coccoides*), *Bacteroides/Prevotella*, *Bifidobacterium*, *Lactobacillus/Leuconostoc/Pediococcus*, *Escherichia coli* (*E. coli*) as well as *Faecalibacterium prausnitzii* (*F. prausnitzii*).

#### Metagenomic sequencing

Intestinal bacterial gene content was determined by high throughput ABI SOLiD sequencing technology of total fecal DNA as described previously by our group [17], an average of 76.5±36.5 (mean ± sd) million 35 base-long single reads were determined for each sample (a total of 393 Gb of sequence). By using corona_lite (v4.0r2.0), an average of 24.8±14.3 million reads per individuals were mapped on the reference catalog of 3.3 million genes [24]–[25] with a maximum of 3 mismatches. Reads mapping at multiple positions were discarded and an average of 14.2±8.1 million uniquely mapped reads per individuals were retained for estimating the abundance of each reference gene by using METEOR software [26]. Abundance of each gene in an individual was normalized with METEOR by dividing the number of reads that uniquely mapped to a gene by its nucleotide length. After that, normalized gene abundances were transformed in frequencies by dividing them with the total number of uniquely mapped reads for a given sample. The resulting set of gene frequencies, termed as microbial gene profile of an individual, was used for further analyses.

#### Identification of patients with low and high gene counts (LGC and HGC, respectively)

The two groups of patients were defined using the 480 000 gene threshold. Genes significantly different in groups of individuals were identified by Mann-Whitney tests using p-value threshold <0.0001. They were clustered by an abundance-based binning strategy, using the covariance of their gene frequency profiles among the individuals of the cohort, as described in the previous study [24]. Abundance of a given cluster in each individual was estimated as a mean abundance of 25 arbitrarily selected 'tracer' genes for each cluster; these values were close to those obtained by using all the genes of a cluster.

### Biochemical measurements

Plasma glucose was measured by hexokinase method (ARCHITECT system, Abbott, Park, Illinois, USA). Plasma insulin was determined by chemiluminescence (ARCHITECT system, Abbott). Plasma triglyceride and free fatty acids (FFA) were measured with Biomérieux kits (Marcy l’Etoile. France), and total cholesterol, high density (HDL), and low density (LDL) lipoprotein cholesterol were measured with Labintest kits (Aix-en-Provence. France). High sensitive (hs) CRP was measured by immuno-nephelometry on an IMMAGE analyzer (Backman-Coulter, Villepinte, France)..Leptin and interleukin-6 (IL-6) were determined by using enzyme-linked immunosorbent assay (ELISA) kit (Quantikine, R&D Systems, Oxford, UK). The limit of detection for leptin was 7.8 pg/ml and the intra- and interassay variability was less than 3.5 and 5.5%, respectively. Adiponectin was determined by using ELISA (Bühlman, Basel, Switzerland). The limit of detection was 0.019 ng/ml and the intra- and interassay variability was less than 5.5%. Serum concentrations of 5 cytokines (eotaxin, vascular endothelial growth factor (VEGF), interferon gamma-induced protein 10 **(**IP10), monocyte chemotactic protein-1 (MCP-1) and macrophage inflammatory protein-1 beta (MIP-1b)) were simultaneously determined by multiplex biochip technology with the human cytokine multiplex panel kits (Bio-Rad, Hercules, CA. USA). A multiplex assay was performed according to the manufacturer’s instructions. Multi-analytic profiling was performed on the Luminex-200 system and the Xmap Platform (Luminex Corporation. Austin. TX. USA). Acquired fluorescence data were analyzed using Exponent software with standard curves obtained from serial dilutions of standard cytokine mixtures. Serum samples were diluted (1∶4). Plasma soluble CD14 (sCD14) was determined by using ELISA (Quantikine, R&D Systems, Oxford, UK). The limit of detection was 125 pg/ml and the intra- and interassay variability was less than 6.5 and 7.5%, respectively and plasma LPS was measured with the Limulus amebocyte lysate kinetic chromogenic methodology that measures the color intensity directly related to the endotoxin concentration in a sample, using Endosafe-MCS (Charles River laboratories, Lyon, France).

### Statistical Analysis

Data were analyzed using SAS 9.2 and SAS Enterprise Guide 4.2 (SAS Institute Inc., Cary. NC. USA) and R version 2.13 Software (http://www.r-project.org). Homeostasis model assessment of insulin resistance (HOMA-IR), insulin sensitivity (HOMA-S%), and b-cell function (HOMA-B%) were calculated by HOMA Calculator from www.dtu.ox.ac.uk/homacalculator. All data are expressed as mean ± SEM. P values ≤0.05 were considered as significant unless specified otherwise. Bonferroni correction was used for the adjustment of the food and nutrient analysis.

### Identification of dietary patterns

Cluster analysis was employed to derive dietary patterns from the data. Overweight and obese subjects were grouped based on similarities in eating behaviors. Prior to this analysis, all food categories were standardized to a mean of 0 and a standard deviation of 1 in order to ensure that quantities consumed were comparable across different categories. A comparison of two clustering methods for use with dietary data (Ward’s Agglomerative Hierarchical Clustering and k-means clustering method) was undertaken as described previously [27]. K-means was found to be the most appropriate method to use in the conditions of the present study. Group membership is determined by a series of steps. The algorithm starts by a set of selected seeds and an estimation of the centroid (the multidimensional equivalent of the mean). Each individual is assigned to the nearest centroid and temporary clusters are formed. The seeds are then replaced by the centroid for each group. The process is repeated until no further changes occur in the groups. The k-means function fits a user-specified number of centers (the parameter k), such as that the within-cluster sum of squares from these centers is minimized, based on the Euclidian distance or another chosen distance. Since the number of clusters must be established *a prior,* several solutions were compared with a varying number of clusters (from 1 to 4).

**Figure 2:**
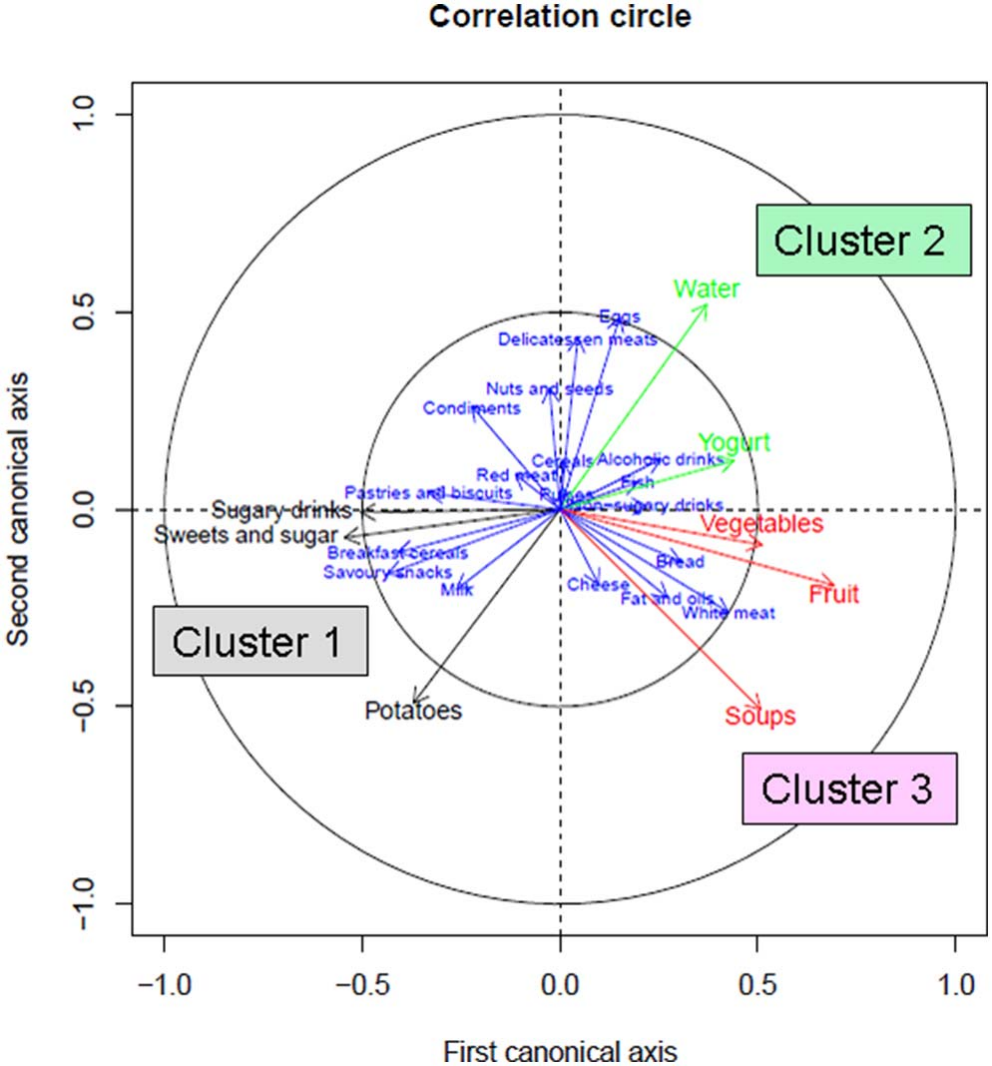
Canonical analysis: graphical representation of the food categories by cluster. A graphical representation of the food categories that created the distinction between the clusters i.e. those which were strongly correlated with canonical axis (Can) or significantly different between clusters (KW test with Bonferroni correction). The can 1 axis separates Cluster1 from 2 or 3, the can 2 axis separates Cluster 2 from 1 or 3. If the food category is strongly correlated with the two canonical axes it separates the three clusters at the same time. Food categories shown in black characterise Cluster 1, in green characterise Cluster 2, and in red characterise Cluster 3. The projection of each food or drink category on each canonical axis represents the contribution of this category to the building of this canonical axis. Therefore, if a category has a high contribution to the first axis (e.g. fruit), it discriminates Cluster 1 from Cluster 2 or Cluster 3. The food categories with weak contribution (below 0.5 in the inner circle) are shown in blue. These categories do not contribute to the discrimination/characterization of the three clusters. The distance between the centre point of the figure and the food category represents the correlation to the canonical axis and therefore the contribution to the separation of clusters. Food categories close to the axis between Clusters 2 and 3 indicate that intakes are similar, as for yogurt. Food category names have been shorted in this figure for readability.

To visualize the food categories that significantly distinguish one pattern from another, a Canonical Discriminate Analysis was implemented through SAS (CANDISC procedure) in order to transform the food categories into two canonical axes that achieve maximum separation between the clusters. The canonical coefficients were used to assess the contributions of food categories to the separation by evaluating their signs and magnitude. Using canonical discriminate analysis for k-means, the 26 food categories were transformed into two canonical variables from the patterns. This approach permitted the visualization of the food categories that significantly distinguish one pattern from another in the overweight/obese subjects (Figure 2). Overweight and obese subjects were classified according to their general dietary pattern. Food or drink categories typically high in fat, sugar or salt and low in other nutrients and fibers were regarded as ‘less healthy’, whereas those typically low in fat, sugar or salt and higher in fiber, fruits and vegetables were regarded as ‘healthier’ [28], [29]. Higher or lower intakes (as appropriate) of such categories contributed to the assignment of a name to a particular group of subjects. The lean subjects were projected onto the clusters of the overweight and obese subjects to examine their distribution (Figure S1 in Supporting Information S1).

### Links between dietary patterns and bioclinical variables including gut microbiota

Since the food and nutrient data were not normally distributed, Kruskal-Wallis tests were used to test for differences between the dietary clusters, while Wilcoxon rank-sum tests were used to test for individual differences between each set of 2 clusters in food and nutrient data. Considering the influence of age on clinical parameters, we performed stratified Kruskal-Wallis tests stratified by age groups (R package = coin) for analyzing the differences in clinical variables as well as gut microbiota between the dietary patterns. When Kruskal-Wallis tests were significant, differences between each set of 2 clusters were assessed with stratified post-hoc Nemenyi tests. The stratified tests for trend (R package = coin) were performed to confirm the trends of clinical markers across the dietary clusters (ordered in terms of healthiness starting with the least healthy).

Partial spearman tests were used to verify the link between gene counts/the 7 bacterial groups and dietary categories/bioclinical variables by adjusting for age. Links between dietary clusters and gene richness groups (low gene count/high gene count: LGC/HGC groups) were investigated using an age-stratified Cochran-Mantel-Haenszel test. Trends were assessed with Mantel-Extension test [30].

Canonical Correlation Analysis was used to maximize the correlation between the set of the food categories that significantly separate the clusters and a set of selected clinical parameters. Two pairs of canonical axes were determined; one pair was from the food categories and one pair from the clinical parameters. The canonical coefficients were used to assess the contributions of each food category and each clinical parameter to the correlation by evaluating their signs and magnitude. This approach permitted to visualize the association between the food categories that significantly distinguish one pattern from another and the selected clinical parameters.

## Results

Overweight and obese subjects were divided into dietary clusters with the number of clusters based on the optimum results obtained using three statistical parameters and practical considerations given the interpretability of the nutritional data. The optimum number of clusters (k) was selected as three (k = 3). Food categories that significantly distinguish one pattern from another in the overweight and obese subjects are presented in Figure 2. The lean subjects were found to be unequally dispersed across the clusters (Figure S1 in Supporting Information S1). The three dietary clusters were then analyzed in relation to clinical and biological factors.

### Subject general characteristics

**Figure 3:**
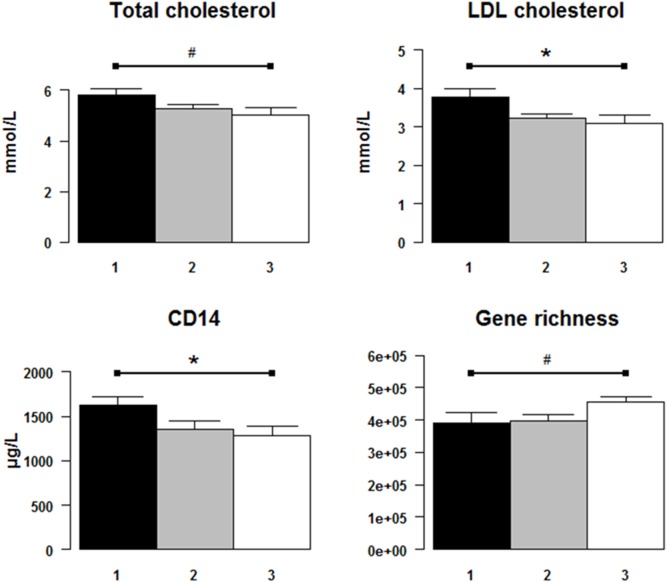
Differences of metabolic and inflammatory markers after stratified Kruskal-Wallis tests in the 3 dietary pattern clusters. Black, grey and white columns represent the median values of the parameters in Cluster 1, Cluster 2 and Cluster 3, respectively, after age adjustment (see Methods S1 in Supporting Information S1). *: significant differences (p≤0.05) between the 3 clusters after stratified Kruskal-Wallis tests, #: a tendency of differences (0.05<p<0.15) between the 3 clusters.

Subject characteristics are shown in Table 1 and Table S1 in Supporting Information S1. In the clusters of overweight and obese subjects, no significant difference was detected in the proportion of women or men, however, mean age of subjects in Cluster 3 was higher (52.2±2.3 years), while mean age of subjects in Cluster 1 was lower (34.4±2.7 years) (p = 0.001 across the 3 clusters). Importantly, body weight and adiposity markers did not differ significantly between the 3 clusters (Table 1). Level of physical activity was also similar across the clusters. There were no differences in plasma glucose homeostasis including surrogate insulin resistance index (HOMA-IR) between the three clusters. A trend towards significance was seen in total- and LDL-cholesterol that were significantly different across the clusters after age adjustment, Cluster 1>Cluster 2> Cluster 3 (Figure 3). Results were significant after stratified tests by age for trend (p = 0.03 for total cholesterol and p = 0.01 for LDL-cholesterol) with the greatest difference seen between Clusters 1 and 3 (when comparing the medians Figure 3) but between cluster 1 and 2 (when comparing the mean values as in table 1).

**Table 1:** Subject Characteristics in the lean and the overweight/obese groups and in the 3 dietary clusters.

	Lean (n = 14 w)	All overweight/obese subjects (n = 45, 39w/6m)	Dietary pattern clusters in overweight/obese subjects			P value	
			Cluster 1 n = 1412w/2m	Cluster 2 n = 1815w/3m	Cluster 3 n = 1312w/1m	Kruskal P*	Trend P**
**Adiposity markers**							
Body weight (kg)	60.81±2.48	91.51±1.98	96.42±3.64	89.67±3.41	86.73±3.14	0.67	0.85
BMI (kg/m^2^)	22.62±0.58	33.2±0.55	33.71±0.98	33.55±0.87	32.91±1.30	0.83	0.54
Total Fat mass (%)	29.68±1.19	39.42±0.94	39.51±1.67	40.22±1.57	39.88±1.61	0.89	0.95
Waist circumference (cm)	75.20±1.80	106.11±1.41	108.68±2.50	104.28±2.45	106.00±2.89	0.30	0.42
Adipocyte diameter (µm)	53.06±1.2	108.8±1.09	108.39±1.83	108.56±2.18	108.71±1.87	0.91	0.81
Leptin (ng/ml)	20.68±3.24	50.68±3.16	57.50±6.14	49.28±4.86	47.22±5.46	0.97	0.84
**Plasma glucose homeostasis and insulin sensitivity**							
Fasting glycaemia (mmol/l)	4.49±0.13	5.22±0.06	5.06±0.05	5.31±0.10	5.28±0.14	0.46	0.47
Fasting insulinemia (µU/ml)	6.27±0.65	8.93±0.62	9.69±1.53	8.69±0.70	7.29±0.72	0.63	0.77
HOMA-IR	0.79±0.09	1.17±0.08	1.25±0.20	1.14±0.09	0.96±0.09	0.64	0.74
Adiponectin (µg/ml)	9.36±1.09	14.13±0.9	15.28±1.69	14.30±1.25	14.40±1.86	0.61	0.37
**Plasma lipid homeostasis**							
Total cholesterol (mmol/l)	4.39±0.2	5.32±0.12	5.52±0.26	5.29±0.13	5.29±0.28	*0.09* ^†^	***0.03***
HDL cholesterol (mmol/l)	1.57±0.1	1.41±0.05	1.37±0.10	1.48±0.08	1.35±0.09	0.87	0.87
LDL cholesterol (mmol/l)	2.51±0.18	3.3±0.11	3.62±0.22	3.22±0.10	3.26±0.26	***0.05*** ^‡^	***0.01***
Triglycerides (mmol/l)	0.68±0.07	1.31±0.12	1.19±0.18	1.28±0.17	1.45±0.29	0.61	0.46
Fasting FFA (mmol/l)	0.62±0.07	0.45±0.02	0.45±0.05	0.45±0.04	0.48±0.05	0.86	0.59
**Inflammatory markers**							
hsCRP (mg/l)	1.45±0.41	3.79±0.44	4.07±0.99	3.64±0.70	3.31±0.67	0.94	0.93
IL-6 (pg/ml)	1.13±0.18	2.08±0.29	2.75±0.74	1.43±0.27	2.06±0.50	0.19	0.55
LPS (pg/ml)	1.57±0.23	2.19±0.21	2.13±0.24	2.00±0.35	2.79±0.55	0.42	0.54
sCD14 (pg/ml)	1358.73±78.92	1390.6±59.57	1607.9±99.1	1350.5±91.4	1263.1±135.5	***0.05*** ^†^	***0.01***
**Adipose tissue macrophages**							
HAM56	2.53±0.35	3.87±0.36	3.35±0.64	4.14±0.62	4.18±0.75	0.50	0.29
HAM56%	7.55±1.1	13.58±1.2	11.07±1.77	14.74±2.23	15.20±2.60	0.48	0.26
CD163	2.35±0.39	1.89±0.22	1.3±0.3	1.7±0.3	2.6±0.4	0.15	***0.05***
CD163%	6.66±1.18	6.67±0.72	4.6±0.9	6.3±1.12	8.7±1.2	0.17	*0.07*
**Chemokines**							
MCP1 (pg/ml)	50.11±8.95	36±3.95	46.55±7.07	34.79±6.99	28.39±7.77	0.41	0.24
VEGF (pg/ml)	82.89±18.74	88.07±13.1	68.10±18.06	102.99±19.69	57.98±14.50	0.31	0.91
Eotaxin (pg/ml)	93.11±17.99	62.95±4.63	55.39±9.59	71.58±8.55	65.56±6.43	0.36	0.38
IP10 (µg/ml)	328.6±32.66	604.09±48.23	635.72±75.40	486.56±64.85	707.05±123.38	0.16	0.93
MIP-1b (pg/ml)	133.4±23.8	134.09±10.8	159.46±27.89	111.26±12.98	119.61±14.12	0.34	0.36

Data are presented as means ± SEM; n = 45 subjects. BMI: Body Mass Index; *Kruskal-Wallis rank sum test stratified by age groups. **tests for trend stratified by age. *P* value ≤0.05 is shown in bold italics. 0.05<*P* value<0.15 is shown in italics. Stratified post-hoc Nemenyi tests stands for variance between each set of 2 clusters: ^†^Significant difference between Clusters 1 and 3;.^‡^Significant difference between Clusters 1 and 2.

As expected, when comparing the whole group of overweight and obese subjects with the lean subjects (Table 1, and Table S1 in Supporting Information S1), overweight and obese subjects had the highest adiposity markers (body weight, fat mass, adipocyte diameter and leptin levels) and perturbed plasma lipids and markers of plasma glucose homeostasis. Of interest, when comparing the different overweight and obese clusters with the lean subjects, Cluster 3 was found to be similar to the lean subjects for insulin sensitivity and plasma FFA, whereas Cluster 1 remained worse than lean subjects and kept the same profile as the overweight and obese subjects.

### Food and nutrient intakes

Mean daily food intakes are shown in Table 2 and Table S2 in Supporting Information S1. When the absolute food intakes were compared between the obese/overweight and lean groups, relatively few differences were observed. Compared to obese/overweight subjects as a whole, in lean subjects the consumption of sugary drinks and water were significantly lower and the intake of vegetables tended to be lower but the intake of fruits was similar. The percentages of consumers for each food category (Table S3 in Supporting Information S1) supported these results, differences were more commonly seen in beverages, e.g. fewer lean subjects were consumers of sugary drinks compared to overweight and obese subjects (57% vs. 82%).

**Table 2:** Mean daily food consumption (grams) for lean, overweight/obese subjects and the 3 dietary clusters.

	Lean (n = 14)	All overweight and obese (n = 45)	Overweight and obese dietary clusters			
Food category			Cluster 1 (n = 14)	Cluster 2 (n = 18)	Cluster 3 (n = 13)	*P* *
Bread and breadproducts	73.81±12.66	75.07±5.54	65.39±8.04	71.61±8.71	90.27±11.67	0.156
Cereals e.g. rice,pasta	135.29±26.53	80.18±7.69	76.29±14.39	87.26±13.78	74.56±11.23	0.876
Pulses e.g. lentils	4.76±4.76	4.28±1.52	3.84±2.37	4.76±2.58	4.07±3.08	0.943
Potatoes includingchips	41.67±13.73	40.56±5.19	66.80±10.69	18.45±4.12	42.91±7.50	***<0.001*** ‡
Breakfast cereals	1.43±1.43	4.00±1.54	10.10±4.22	1.90±1.48	0.33±0.33	0.026
Milk	60.95±21.69	98.44±17.54	149.69±39.75	68.76±23.19	84.34±25.59	0.185
Yogurt**	121.79±21.97	95.49±14.47	31.58±9.80	121.59±23.58	128.19±30.34	***0.0016***
Cheese	50.95±19.07	31.84±4.39	31.12±5.19	26.31±5.46	40.29±12.03	0.635
White meat e.g.chicken	19.52±6.69	38.69±4.26	26.45±7.31	33.94±4.26	58.46±9.14	0.010
Red meat e.g.beef, lamb	54.76±10.38	54.48±4.76	55.63±8.82	56.18±8.29	50.88±7.76	0.841
Delicatessen meatse.g. ham	46.43±15.61	42.84±5.27	32.55±5.59	60.03±10.05	30.11±7.79	0.017
Fish and fishproducts	53.33±15.01	30.08±3.86	22.55±7.26	33.06±6.09	34.07±6.86	0.342
Fruit	203.81±36.14	206.44±24.16	79.12±22.15	191.32±34.98	364.51±29.57	***<0.001*** †
Vegetables	115.00±19.69	175.24±15.76	107.42±19.91	181.52±21.77	239.57±31.60	0.003†
Fats and oils	22.50±2.40	20.07±1.46	17.48±2.68	18.36±1.82	25.23±3.00	0.101
Eggs and eggdishes	11.43±5.01	19.84±3.74	9.08±2.83	32.62±7.63	13.74±4.60	0.070
Sweets, confectionaryand table sugar	19.17±5.08	48.71±9.36	96.19±20.32	35.49±12.40	15.88±4.04	***<0.001***
Pastries and sweetbiscuits	40.43±10.98	53.80±8.82	78.64±23.92	51.24±8.67	30.60±8.48	0.169
Soups	107.14±29.99	40.87±8.70	18.88±6.16	13.10±4.98	103.02±20.34	***<0.001*** † §
Savoury snacks, piesand pizzas	40.00±14.51	19.90±4.58	39.59±10.72	12.37±5.86	9.12±3.70	0.132
Condiments and sauces	5.00±2.81	12.87±2.25	14.80±3.07	17.17±4.73	4.84±1.15	0.077
Nuts and seeds	0.48±0.48	1.30±0.56	0.82±0.63	2.54±1.26	0.11±0.11	0.451
Water (all types)	384.52±62.23	774.14±59.26	463.91±81.93	1047.94±75.94	729.12±89.26	***<0.001*** ‡
Drinks without sugarwithout alcohol e.g. tea, coffee	475.00±46.12	271.26±27.40	232.55±69.43	278.94±31.93	302.31±41.05	0.274
Drinks with sugarwithout alcohol e.g.soda, fruit juice	46.43±15.45	162.42±31.40	300.97±77.34	139.26±34.40	45.27±16.88	***0.0016*** †
Drinks with alcohole.g. wine, beer	39.76±16.42	33.06±7.98	14.29±6.41	40.71±13.93	42.69±18.26	0.298

Data are presented as means ± SEM.

*Kruskal-Wallis rank sum test with Bonferroni correction, *P* value significant at ≤0.002 is shown in bold italics. Wilcoxon rank sum test stands for variance between overweight and obese individual clusters.

† significant difference between Clusters 1 and 3.

‡ significant difference between Clusters 1 and 2;

§ significant difference between Clusters 2 and 3. *P* values testing variance between lean subjects and all overweight and obese subjects and between lean subjects and the individual clusters are shown in Table S2 in Supporting Information S1.

**This group contains other fermented dairy products, e.g. fromage blanc.

We observed significant differences in intakes across the 3 clusters for particular food groups. In obese/overweight subjects, Cluster 1 was characterized by the highest consumption of potatoes, sweets, confectionary and table sugar and sugary drinks and the lowest consumption of fruits, yogurt and water. This pattern appeared to have the least healthy eating behavior compared to the other patterns. Inversely, Cluster 3 was characterized by the highest consumption of fruits, yogurt and soups (likely to be composed mainly of water and vegetables) with a lower consumption of sweets, confectionary and table sugar, and sugary drinks. Intake of vegetables was also a key in characterizing this cluster and mean intakes showed a trend to significance. Cluster 3 appeared to have the healthiest eating behavior compared to the other two clusters. Cluster 2 was characterized by the highest consumption of water and a consumption of yogurt similar to that of Cluster 3. In terms of healthfulness it was in-between Clusters 1 and 3. Compared to food consumption in the lean subjects, we observed some similarities between dietary patterns of lean subjects, and overweight and obese subjects grouped in Cluster 3 (the healthiest cluster), the latter even tended to consume more fruit and vegetables. On the contrary, overweight and obese subjects grouped in Cluster 1 had a higher consumption of sweets, confectionary and table sugar and sugary drinks compared to the lean ones (Table 2 and Table S2 in Supporting Information S1).

Tables S4 and table S5 in Supporting Information S1 show the nutrient intakes of the 3 clusters. Surprisingly, there were no differences in total energy intake across the clusters. However protein intake expressed as percentage of energy was lower in Cluster 1. Higher micronutrient intakes were generally observed in Cluster 3, while lower intakes were found in Cluster 1, supporting the characterization of the groups in terms of healthfulness according to food consumption. Total fiber intake was also highest in Cluster 3 and lowest in Cluster 1.

### Dietary patterns, systemic and adipose tissue inflammation

As expected, overweight and obese subjects had increased circulating hsCRP and IP10 and increased percentage of HAM56+cells in adipose tissue compared to lean subjects. The obese subjects grouped in the healthy Cluster 3 had similar circulating levels of IL6 and even better levels of MCP1 compared to lean subjects.

In the overweight/obese subjects, Cluster 3 had the lowest level of a systemic marker of inflammation, plasma sCD14, followed by Cluster 2 and then Cluster 1 (Figure 3, Table S1 in Supporting Information S1). However, the other measured systemic inflammatory markers (such as hsCRP, IL6, LPS) and the number of macrophages stained with HAM56 in adipose tissue were not significantly different among the 3 clusters. Interestingly this trend toward a less systemic inflammatory phenotype (at least for sCD14) was associated with an increased number of M2-alternatively activated macrophages in adipose tissue stained with CD163 surface markers (p = 0.05 by stratified tests for trend) as well as CD163% (p = 0.07 by stratified tests for trend) (Table 1).

**Figure 4:**
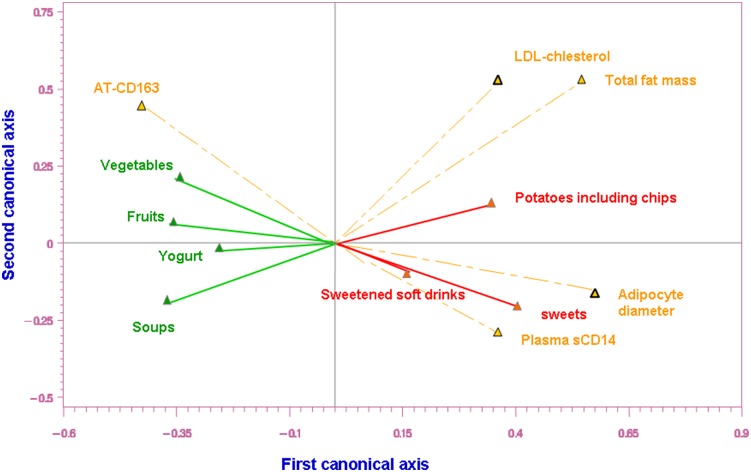
Canonical correlation analysis for significant food categories and selected clinical parameters (all subjects). Visualization of the association between the food categories that significantly distinguish one pattern from another and selected clinical parameters. Pairs of canonical axes were determined to maximize the covariance between the food categories and the clinical parameters. The canonical coefficients were used to assess the contributions of each food category and each clinical parameter to the correlation by evaluating their signs and magnitude. The healthy foods (yogurt, soups, fruits, vegetables) are in the area of CD163+ macrophages indicating the higher the consumption of these healthy foods, the higher the value for the alternatively (M2)-activated macrophages. The less healthy foods (potatoes, sweetened soft drinks, sweets) are in the area of LDL cholesterol, inflammatory parameters CD14, total fat mass and adipocyte diameter indicating that the higher the consumption of these foods, the higher the value of these clinical parameters; Food and clinical parameter arrows pointing in the same direction indicate positive correlation between them. The closer the food is to the clinical parameter, the greater the link (but in some cases this link is not strong, and the value for the correlation is less than 0.05).

These results were confirmed when the food groups that distinguished the clusters were combined and analyzed in relation to selected clinical parameters (Figure 4). When all overweight/obese subjects were analyzed together, a positive association was found between the consumption of fruits, vegetables, yogurts and soups and the amount of adipose tissue macrophage cells stained with (CD163, anti-inflammatory marker) together with a negative link with some adiposity markers (total fat mass, adipocyte diameter), a cardiovascular risk marker (LDL-cholesterol) and a systemic inflammatory marker (sCD14). Greater intakes of potatoes, sweets and sweetened soft drinks are associated with increasing levels of these markers and decreasing adipose macrophages positive for anti-inflammatory markers. These links could not be clearly illustrated in the lean subjects due to the limited fluctuation of normal values in a narrow zone.

### Dietary patterns and gut microbiota

#### Dietary patterns and dominant fecal bacteria

There were no differences in the seven bacterial groups detected by qPCR method among the 3 dietary clusters of overweight/obese subjects (Table S6 in Supporting Information S1). However the overweight/obese group as a whole had lower levels of *Clostridia leptum*, *Clostridia coccoides* and *Bacteroides/Prevotella* groups than the lean subjects (Table S7 in Supporting Information S1). A negative association was found between the Lactobacillus/Leuconostoc/Pediococcus group and cereals intake (e.g. rice, pasta) (Table S8 in Supporting Information S1).

#### Dietary patterns and microbial richness (HGC and LGC)

The differences in total gene counts did not reach statistical significance, but showed a tendency for higher gene richness in Cluster 3 (p = 0.09 by stratified Kruskal-Wallis tests and p = 0.06 by stratified tests for trend) (Figure 3, Table S9 in Supporting Information S1). When splitting subjects in function of gut microbial richness into HGC and LGC as previously described in Cotillard et al (17), the relationship became significant (p = 0.045 by Cochran-Mantel-Haenszel test and p = 0.035 by stratified test for trend). Thus, subjects in Cluster 3 had the highest gene richness and diversity in their gut microbiota. We also used partial Spearman correlation tests to check for links between microbial gene counts and dietary categories by adjusting for age. Consistently, there was a significant positive link between total bacterial gene counts and fruits (rho = 0.43, p = 0.002), as well as between gene counts and soups (rho = 0.35, p = 0.017).

## Discussion

We were able to identify in overweight/obese subjects three distinct dietary clusters and explore differences in host inflammatory variables and gut microbiota in function of the three dietary clusters in a relatively small number of subjects. Those subjects in Cluster 3 were identified as having a healthier dietary pattern, while subjects in Cluster 1 had the least healthy dietary pattern. Associations between the healthfulness of the dietary pattern and LDL-cholesterol, some adiposity markers and inflammatory profile were found in the overweight/obese subjects.

In our study the healthier dietary pattern was characterized by a higher consumption of fruits and vegetables and a lower consumption of confectionary and sugary drinks. These foods have been linked to a healthy dietary pattern in other epidemiological studies in different populations [10]. Consumption of soups was associated to the healthier dietary pattern, a result to be expected given its composition which is likely to be mainly of water and vegetables. Greater low-fat fermented dairy product intake, largely driven by yoghurt intake, was found to be associated with a decreased risk of type 2 diabetes development in prospective analyses [31], [32], but a null association was found between high fat dairy intake and disease development. In the present study, the yogurt group contained other fermented dietary products (such as fromage blanc) and included both low and high fat yogurts. Our results showed that the consumption of yogurt was also often associated to the healthier dietary pattern.These results merit further investigation.

When looking to the impact of dietary patterns on metabolic health, many prospective studies have demonstrated the association between dietary patterns and weight changes in longitudinal studies over 9 years [4] or during whole adult life [6] using a semi quantitative food-frequency questionnaire. Food groups have been suggested to predict short-term weight changes as in the EPIC-Potsdam Cohort [7]. However, many inconsistencies exist in those studies [5]. Increased consumption of vegetables and fruits in the diet was not found to be effective for weight loss. A 100g fruit and vegetable intake was associated with only a −14 g weight loss per year in a multi-center European study (DIOGenes) [33]. Increasing fruit and vegetable intake was found to reduce risk of weight gain only in people susceptible to weight gain with smoking cessation [33], [34]. In the present study, Cluster 3 which had an overall healthier dietary pattern containing more fruits and vegetables was found to have no impact on classifying weight-stable subjects according to body weight or other adiposity markers.

In the present study, fiber intake was higher in Cluster 3 compared with the other clusters. Higher fiber intake was demonstrated previously to be associated with lower weight gain [35]. Despite the higher fiber intake observed in Cluster 3, body weight and total energy intake were not significantly different across the 3 clusters. Interestingly, in the whole group of overweight and obese subjects, lower total fat mass and smaller adipocyte diameter were associated with increased consumption of foods found in Cluster 3 (fruits, vegetables, yogurts and soups); while higher total fat mass and larger adipocytes showed a positive link with increasing potatoes, sweet, and sweetened soft drink consumption. These links could not be demonstrated in the lean group. This might be likely due to the fluctuation of normal values in a narrow zone.

While the identified dietary patterns did not differ in terms of total energy intake or body weight, Cluster 1, the least healthy dietary pattern was associated with the worst metabolic profile demonstrated by a trend toward higher levels of total- and LDL-cholesterol. This trend paralleled the positive links observed between LDL-cholesterol and increasing consumption of potatoes, sweets and sweetened soft drinks, and the negative association with increasing the consumption of fruit, vegetables, and soups but also yogurt, in the overweight/obese group as a whole. These results obtained even with a small number of subjects, are in line with large cohort studies in which healthy dietary patterns have been associated with a lower risk of coronary artery diseases: Health Professional Follow-Up Study [36] and the Nurses Health Study [37].

The three different dietary patterns were associated differently to the inflammatory markers. While no differences were found on hsCRP, IL-6 or LPS levels, Cluster 3 had the highest level of CD163+macrophages anti-inflammatory cells) in adipose tissue and the lowest level of a systemic inflammatory marker (sCD14). Firstly, the subpopulation of CD163 positive cells refers classically to alternatively (M2)-activated macrophages with some anti-inflammatory properties. Bariatric-surgery induced weight loss is associated with increased amount of these CD163+ cells [38] and the amount of CD 163+ cells is associated with drug-induced improvement of insulin sensitivity in clinical studies [39]. In addition, genetically modified mice, with impaired M2 macrophage activation, are prone to diet-induced obesity and insulin resistance [40]. Therefore, the increased adipose tissue anti-inflammatory marker might have a positive impact on health.

Secondly, since CD14 acts as a co-receptor (along with the Toll-like receptor TLR 4 and MD-2) for bacterial LPS [41], the low levels of sCD14 might be associated with a decrease in LPS, one of the principal components of gram-negative bacterial membrane. However, in the present study, no difference was detected in LPS or in the amount of the 7 bacterial groups determined across the 3 clusters. However, the variation in dietary patterns between the 3 clusters was linked to different levels of plasma sCD14 and anti-inflammatory macrophages in adipose tissue subjects. This hypothesis is strengthened by the negative correlations found between these dietary patterns and plasma sCD14, and the positive ones found between these dietary patterns and the anti inflammatory phenotype of adipose tissue.

In spite of the absence of differences between the 3 dietary patterns in the 7 components of gut microbiota, we are not able to rule out the possible role of gut microbiota in linking dietary patterns to changes in inflammation and some metabolic markers. However, a negative association was found between the Lactobacillus/Leuconostoc/Pediococcus group and cereals intake (e.g. rice, pasta) in the whole population. Further studies are needed to understand such link. Additionally, gut microbiota of subjects in Cluster 3 showed higher gene richness than the other clusters. Consistently, this gene richness was also positively associated with fruit consumption. These results are strengthened by a recent study in a European cohort confirming the association between gene richness and fruit and vegetable consumption [24]. We require now a more in-depth knowledge to discover the functional significance behind these links.

We are aware that these results have been found in a limited number of subjects for which there is a high female to male ratio. Identifying dietary patterns presents challenges in any population, especially a small sized one. The validity of the dietary pattern analysis depends on the dietary assessment method itself and the completeness and accuracy of the dietary data collected in the study. Indeed, the analysis of dietary patterns requires decisions, judgments and interpretations which may result in biased results, for example the creation of food categories, and the decisions regarding the number of clusters [3]. Dietary recording in the lean subjects differed from that of the overweight/obese subjects (3 vs 7 days) and compliance with recording might decrease in line with the number of diary days. Furthermore, misreporting of the dietary data may also attenuate any diet and disease relationships and affect any conclusions that can be made [42]. However, there is no reason to believe that the level of misreporting would differ significantly across these subjects with controlled dietary data collection.

Given the results in their entirety, the scenario might be as follows: small differences in dietary patterns can influence the microbial gene diversity and quantity of certain gene classes/species that might have in part a direct impact on some host metabolic (total-cholesterol and LDL cholesterol) and inflammatory markers (sCD14) and accumulation of some subpopulations of adipose tissue macrophages. Additionally, dietary patterns might have also a direct influence on host markers.

To our knowledge this study is the first to explore the impact of dietary patterns on gut microbiota (including seven dominant bacteria and microbial gene richness generated by next-generation sequencing method) and host inflammation in overweight/obese subjects without any dietary transition. This study gives insight into how different dietary patterns might be linked with gut microbiota and host metabolism and inflammation. The variation in metabolic health among overweight and obese individuals might be partially attributable to habitual diets. The results show that the subgroup that follows a healthier diet (in terms of higher consumption of vegetables and fruit, and lower consumption of sweets) shows less pronounced metabolic impairment, even when body weight and total energy intake do not differ from the other groups. This suggests that a healthy eating pattern may provide protection towards development of metabolic and inflammatory diseases even when it has no impact on body weight. Further studies are needed to elucidate the underlying mechanisms that may provide new diagnostic and therapeutic strategies to treat and prevent metabolic diseases.

## Supporting Information

Checklist S1 **CONSORT checklist.**
(PDF)

Protocol S1 **Trial protocol.**
(DOC)

Supporting Information S1 **Supporting files. Figure S1**, Discriminate canonical analysis (graphical representation of the separation of overweight or obese clusters) with lean subjects projected on the representation. **Methods S1,**Visualization purposes for Figure 2. **Table S1,** *P* values for variance in clinical parameters between lean subjects and all overweight/obese subjects, and between lean subjects and individual clusters. **Table S2,** *P* values for variance in food consumption between lean subjects and all overweight/obese subjects, and between lean subjects and individual clusters. **Table S3,** Percentage of consumers for each food category for lean and overweight and obese subjects. **Table S4,** Mean daily nutrient intakes for lean, overweight/obese subjects and dietary clusters. **Table S5,** *P* values for variance in nutrient intakes between lean subjects and all overweight/obese subjects, and between lean subjects and individual clusters. **Table S6,** Differences in gut microbiota (qPCR) in lean, overweight/obese subjects and in the 3 dietary clusters. **Table S7,** *P* values for variance in gut microbiota (qPCR) between lean subjects and all overweight/obese subjects, and between lean subjects and individual clusters. **Table S8,** Correlations between the 7 gut bacterial groups measured by the qPCR and the intake of food groups without stratification to clusters. Heatmap of correlations between qPCR data and food categories. **Table S9,** Differences in gene richness group (LGC/HGC subjects) in the 3 dietary clusters.
(DOC)
