# Supplementary material for: Dietary Patterns Differently Associate with Inflammation and Gut Microbiota in Overweight and Obese Subjects
Source: PLoS One. 2014 Oct 20;9(10):e109434. doi: 10.1371/journal.pone.0109434 (PMC4203727; doi:10.1371/journal.pone.0109434)
Supplement: Protocol S1 — Trial protocol. (DOC) [file pone.0109434.s002.doc]

**BIOMEDICAL ReSEArch PROTOCOL**

**Human Intestinal Microbiome in Obesity and Nutritional Transition**

**MICRO-Obes**

**VERSION N° 2 of 02/07/2008**

**Coordinating Investigator:**

Pr Karine Clément

**CRNH-Ile de France, INSERM U 872 (Eq. 7)**

Pole d’endocrinologie (Service Nutrition)

Pitié-Salpêtrière

83 Bd de l’hôpital

750013 Paris

**Tel : 01 42 34 84 52**

**Fax : 01 40 51 00 57**

**Email :** [karine.clement@pls.aphp.fr](mailto:karine.clement@pls.aphp.fr)

**Co- Investigator**

Dr Salwa Rizkalla

**CRNH-Ile de France, INSERM U 872 (Eq 7)**

Pole d’endocrinologie (Service Nutrition)

Pitié-Salpêtrière

83 Bd de l’hôpital

750013 Paris

Tel 01 42 17 79 81

Email [salwa.rizkalla@pssl.aphp.fr](mailto:salwa.rizkalla@pssl.aphp.fr)

**Sponsor :** Laboratoire CEPRODI - KOT

161, rue de Courcelles

75017 Paris

**Location of Clinical Trial**

Pôle d’Endocrinologie, Centre de Recherche en Nutrition Humaine Ile de France (Pr Basdevant) INSERM U872 (Eq.7), Pitié-Salpêtrière, 83 Bd de l’hôpital, 75013 Paris

Overview

| Visits | V1  Inclusion | V2  **J0-Reg-HC** | V3  **J7-Reg-HC** | V4  J42-**RHC**  **J0 - RST** | V5  **J84 –RST** |
| --- | --- | --- | --- | --- | --- |
| Days | -14 à -1 | 0 | 7±1 | 42±4 | 84±10 |
| Inclusion/exclusion criteria | X | X |  |  |  |
| Study intervention |  | x | x | x | x |
| Signed informed consent |  | x |  |  |  |
| TA | × | x |  | × | x |
| Weight (height) | × | x | x | × | x |
| Electrical impedance |  | x |  | x | x |
| Waist circumference |  | × | x | x | x |
| **Phenotype and nutritional assessment**  - Anthropometric measurements including body composition (DEXA)  - Nutrition diary | x | x    x  x | x  x | x  x  x | x  x  x |
| **Metabolic assessment**  - Glycemia  - Insulinemia  - Glucose tolerace test  - Cholesterol, LDL-, HDL- Cholesterol, TG | x  x | x  x  x |  | x  x  x | x  x  x |
| **Inflammation assessment**  - CRP  - SAA  - IL6  -TNFα |  | x  x  x  x |  | x  x  x  x | x  x  x  x |
| **Adipokine assessment**  - Leptin  - Adiponectin |  | x  x |  | x  x | x  x |
| **Serum bank ( 7 ml)** |  | × |  | x | x |
| Subcutaneous adipose tissue needle biopsy |  | x | x | x | x |
| **Fecal assessment** |  | x |  | x | x |

RHC : Hypocaloric diet

RST : Weight stabilization diet

**SUMmary**

[1 Justification de l’essai – Prérequis 5](#__RefHeading___Toc180407148)

[2 Protection des personnes 8](#__RefHeading___Toc180407149)

[a. Justification éthique du protocole 8](#__RefHeading___Toc180407150)

[b. Dispositions éthiques et réglementaires 8](#__RefHeading___Toc180407151)

[c. Comité de Protection des Personnes (CPP) 8](#__RefHeading___Toc180407152)

[d. Assurance et financement 8](#__RefHeading___Toc180407153)

[e. Modalités de recueil du consentement des personnes 8](#__RefHeading___Toc180407154)

[f. Indemnités 9](#__RefHeading___Toc180407155)

[3 Objectifs 9](#__RefHeading___Toc180407156)

[4 Plan expérimental 9](#__RefHeading___Toc180407157)

[a. Type d’essai 9](#__RefHeading___Toc180407158)

[b. Catégorie de recherche : 10](#__RefHeading___Toc180407159)

[Etude physiologie 10](#__RefHeading___Toc180407160)

[c. Exposé du plan expérimental 10](#__RefHeading___Toc180407161)

[5 Critères d’évaluation 11](#__RefHeading___Toc180407162)

[6 Sélection des personnes 11](#__RefHeading___Toc180407163)

[a. Population étudiée participante: 11](#__RefHeading___Toc180407164)

[i. Durée de la participation des personnes à la recherche : 11](#__RefHeading___Toc180407165)

[ii. Description des personnes participantes : 11](#__RefHeading___Toc180407166)

[iii. Nombre de personnes à inclure : 12](#__RefHeading___Toc180407167)

[b. Recrutement et sortie de l’essai 12](#__RefHeading___Toc180407168)

[7 Réalisation pratique du protocole 12](#__RefHeading___Toc180407169)

[a. Description détaillée des actes pratiqués sur les personnes: 12](#__RefHeading___Toc180407170)

[b. Description de l’organisation logistique générale de l’essai 14](#__RefHeading___Toc180407171)

[c. Calendrier prévisionnel de l’essai (Flow-Chart) 14](#__RefHeading___Toc180407172)

[8 Administration de produits 14](#__RefHeading___Toc180407173)

[9 Dispositifs médicaux 14](#__RefHeading___Toc180407174)

[10 Collection d’Echantillons biologiques d’origine humaine 14](#__RefHeading___Toc180407175)

[11 vigilance de l’essai 15](#__RefHeading___Toc180407176)

[a. Définition des évènements indésirables graves 15](#__RefHeading___Toc180407177)

[b. Procédure à suivre en cas d’événement indésirable grave 15](#__RefHeading___Toc180407178)

[c. Suivi des patients en cas de survenue d’un évènement indésirable grave 16](#__RefHeading___Toc180407179)

[d. Faits nouveaux 16](#__RefHeading___Toc180407180)

[e. Comité de surveillance indépendant 16](#__RefHeading___Toc180407181)

[12 Recueil et traitement des données 16](#__RefHeading___Toc180407182)

[13 Analyse statistique des données 16](#__RefHeading___Toc180407184)

[14 Communication 17](#__RefHeading___Toc180407186)

[15 modifications substantielles du protocole 17](#__RefHeading___Toc180407187)

[16 Confidentialité 17](#__RefHeading___Toc180407189)

[17 Assurance Qualité 17](#__RefHeading___Toc180407190)

[18 REFERENCES BIBLIOGRAPHIQUES 18](#__RefHeading___Toc180407191)

[19 Annexes Erreur ! Signet non défini.](#__RefHeading___Toc180407192)

[1. Formulaire d’information et de consentement **Erreur ! Signet non défini.**](#__RefHeading___Toc180407193)

[2. Copie de l’attestation d’assurance **Erreur ! Signet non défini.**](#__RefHeading___Toc180407195)

[3. Copie de l’avis favorable du CPP : **EN ATTENTE**](#__RefHeading___Toc180407196)

[4. Déclaration d’Helsinki](#__RefHeading___Toc180407198)

[5. Lieu de réalisation de la recherche **Erreur ! Signet non défini.**](#__RefHeading___Toc180407200)

[6. Justificatif de paiement taxes AFSSAPS **Erreur ! Signet non défini.**](#__RefHeading___Toc180407201)

# Justification of test 1 – requirements

This is a clinical protocol in the framework of the **Programme de l’Agence Nationale de l’Alimentation (ANR) 2007-2010 (**French National Food Agency (ANR)) with INRA: MICROBIAL GENOMICS LARGE SCALE - GMGE; coordinator: Joël Doré. The project is selected in the framework of a Franco-Chinese agreement between the ANR and the Ministry of Science and Technology of China (MOST). It will be in association with a Chinese project.

**1. 1 – CONTEXT AND STATE OF THE ART**

**Obesity and nutrition transition.**

The nutrition transition observed over several decades in Western countries and more recently emerging (such as China) has been accompanied by an obesity epidemic and increased metabolic (insulin resistance, dyslipidemia) and cardiovascular complications. According to figures from WHO (www.who.int), about one billion adults are overweight (BMI> 25kg / m²), of which 300 million are obese (BMI> 30kg / m²). In France, the prevalence of obesity is now 12.4% (against 8.2% in 1997) in the population over 15 years. Severe obesity is rarely associated with a genetic cause (1) (2).

Complex interactions between susceptible genes and environmental factors are the most common causes of obesity. Among these factors, the nutritional transition contributes significantly to alterations in the regulation of the functioning of adipose tissue (increased release of fatty acids, secretion of adipokines ...) and its relationship with other tissues critical for weight control (liver, muscle, pancreas and brain). Obesity is now recognized as a state of chronic low grade inflammation, characterized by the increase of systemic inflammatory markers. These markers exert, in addition an pro or anti-inflammatory, immune and metabolic functions that are related in part to the accumulation of adipose tissue in the circulating macrophages, with hyperplasia and hypertrophy of the adipocytes (3-5) (6, 7)

Even though some but not all environmental factors have been defined, the epidemic of obesity appears virtually impossible to control for the time being, and the mechanisms associated with fat mass expansion need to be urgently identified (Hill et al 2003).

Recently studies in rats and humans suggested a potential role of the human intestinal microbiota in energy harvest from food. This new paradigm opens perspectives for recommendations and dietary adaptations that may contribute in tackling this major problem of public health.

**Human intestinal microbiota**.

The human intestinal microbiota has many functions, most of which are beneficial to the host. In turn, homeostasis of the intricate interactions between food, microbiota and host is essential to the maintenance of health and well being ; and conversely, the disruption of any constituent is susceptible to lead to perturbations and digestive pathologies (functional, inflammatory or infectious..). The importance of the intestinal microbiota and the production of lipopolysaccharide (LPS) in the inflammatory diseases and metabolic syndrome has been demonstrated in rodents (8-10).

The relationship between changes in the intestinal bacterial flora, the inflammatory status and adipose tissue has not yet been explored in humans. Emerging chronic diseases and could be related to alterations in the composition of the microbiota (dysbiosis).

**Microbiota and weight gain.** In 1983, Wostmann et al observed that germ-free rodents required 30% more calories than conventional animals to maintain body-mass. Mechanisms allowing to explain this observation remained elusive until recent studies of the group of Jeffrey I. Gordon that suggest a role of the intestinal microbiota in energy harvest from food [Bäckhed et al., 2005, Sonnenburg et al., 2005] and in regulation of fat storage mechanisms [Bäckhed, 2004]. It was shown that 8 weeks old germ-free mice eat more, exercise less but have a lesser volume of fat tissues than their conventional (normally colonized) counterparts. Within two weeks, ex germ-free mice associated with a microbiota had a 20% increase in body-fat (DEXA measurements) and emerging insulin-resistance in spite of a 30% decrease

in food intake [Backhed, 2004]. No change in gene expression for Acetyl-CoA carboxylase, FAS, aP2 or PPAR-gamma were observed but the activity of lipoprotein lipase was increased. Comparison of ex germ-free mice associated with the microbiota of lean *versus* obese (ob/ob) mice showed that the transfer of the “obese” microbiota induced a more efficient energy harvest from the diet [Turnbaugh et al 2006.Nature]. This was correlated with a dysbiosis in the obese mice faecal microbiota characterized by an increased representation of the phylum Firmicutes and a reduced representation of the phylum Bacteroidetes [Ley et al., 2005].

These results from animal studies were completed by two independent human studies. Comparison of the microbiota composition between lean and obese humans and its evolution in obese patients loosing weight during a low calories or low fat intervention indicated a lesser proportion of Bacteroidetes and increased Firmicutes in obese subjects that tended to revert to that of lean controls upon low calory dietary intervention [Ley et al 2006]. Dynamics of microbiota composition and bacterial fermentation products in obese patients upon low calory intervention showed a reduced concentration of butyrate and lesser proportions of the *Roseburia*-*Eubacterium rectale* subgroup (Phylum Firmicutes) and

*Bifidobacterium* genus as obese patients were losing weight [Duncan et al 2007]..

**Bacterial**

**colonization**

**LPL activity**

**in adipocytes**

**storage of triglycerides**

**in liver and adipocytes**

**hepatic**

**lipogenesis**

**fiaf gene**

**in**

**epithelium**

**oxydation**

**of fatty acids**

**degradation of**

**dietary**

**polysaccharides**

In man as in rodents, the composition of the microbiota seems to determine the efficacy of energy harvest from food, by mechanisms that could influence weight gain or weight loss. Germ-free animals are protected against diet-induced obesity and the impact of the microbiota on the efficacy of calory harvest from the diet has been associated in part with the regulation of specific host genes at the level of intestinal epithelial cells. Target eukaryote signalling molecules modulated by the microbiota in mice included Resistin-like molecule Beta (RelmB) and Fasting induced adipose factor (Fiaf), both increased upon conventionalization (He Gastroenterology 2003 ; Backhed PNAS 2007). RelmB acts as a proinflammatory immuno-cytokine but also as a hormone involved in body-weight control (Sholima

Diabetologia 2005). Fiaf 1) acts as an inhibitor of lipoprotein lipase, thereby increasing storage of triglycerides in adipocytes and 2) increases fatty acid oxidation by reduction of Cpt1, Mcad, Pgc1alpha gene expression (Backhed PNAS 2007). In turn, Fiaf-KO mice lose protection against fat storing activity and gain weight.

The gut microbiota is an essential environmental factor that regulates fat storage. Nevertheless, the signalling molecules of bacterial origin have yet to be identified

**Human intestinal metagenomics**.

Metagenomics is a relatively new and powerful approach to study the genomes of all microbes in an ecological niche. The genomic content of our resident intestinal microbial community is largely unknown due to the inability to culture the vast majority of its dominant microbes. In metagenomics, the microbial population is extracted from its environment, the DNA is purified, and, using high throughput shotgun cloning techniques, the entire sample is sequenced. From such data, the repertoire of genes of both cultured and yet uncultured microbes can potentially be deduced. This permits the design of generic tools for massive microbiomic profiling of the intestinal ecosystem. In addition, large metagenomic inserts can be subjected to a functional investigation, giving access to yet totally unexplored biological resources. Beyond its innovative character, the usefulness of metagenomics has already been well documented (Reisenfeld, 2004 ; *The new science of metagenomics : revealing the secrets of our microbial planet*, National Research Council of the National Academies, The National Academies Press, Washington DC. 2007).

Initial developments applying metagenomics to the human intestinal microbiota have been limited to sequence-based exploration of the biodiversity of the microbial communities (Manichanh et al 2006, Eckburg et al 2006) or the gene repertoire of only two individuals (Gill et al 2006). The functional metagenomics strategy has so far been limited to soil (Williamson et al AEM 2005) and animal intestinal environments (Walter et al AEM 2005; Beloqui et al JBC 2006). We have only just begun to apply it to the human intestinal ecosystem (Gloux et al, 2007).

**1.2 – *Project aims AND INNOVATIVE ASPECTS***

**Project objectives: scientific and socio-economic**

The global obesity epidemic imposes a huge and rapidly growing challenge for public health services. The **overall aim of MICRO-Obes** is to investigate the possibility of a significant relationship between nutritional and metabolic status on the one hand, and the gut microbiota on the other hand, thereby opening novel avenues to understand, prevent or reverse this condition. To reach this overall aim, **MICRO-Obes** has the following objectives:

- Scientific and technological objectives :
- Sequence the dominant gene repertoire of the human intestinal tract and identify sequence signatures of health/obesity
- Develop high-throughput methodologies for metagenomics profiling
- Define the impact of nutritional transition on the intestinal microbiota and identify metagenomics signatures of nutritional transition
- Identify functions involved in host-microbe interactions with relevance to obesity
- Socio-economic objectives :
- Diagnostics : Develop tools to analyze metagenomic data. The novel strategies proposed for the analysis of the human intestinal microbiota in health and diseases combine several advantages: they are high throughput, near exhaustive by the depth of exploration that will determine the sensitivity of the method, and they combine assessment of composition with that of the genetic makeup and hence functional potentials of the ecosystem.
- Nutritional recommendations: Provide strategic information for the adaptation of nutritional recommendations to patients based on intestinal microbiome characteristics. This will be made possible by the identification of microbial signatures of obesity and nutritional transition and by exploration of the functional meaning of the observed Dysbiosis.
- Functional foods: Provide knowledge-based recommendations for novel strategies to prevent obesity. The knowledge of deviations associated with disease will be most valuable to define strategies aiming at restoring a microbiota as close as possible to that of healthy individuals.

**1.3 - Originality/novelty within the project**

The project is based on the application of the metagenomics approach, both in its sequence-based and function-based developments. The metagenomics approach will allows to clone, sequence and describe the functional gene core - c.a. the repertoire of genes - of the intestinal microbiota of healthy individuals and obese patients, irrespective of the culturability of the source-microorganisms, which is essential since over 80% of dominant human intestinal bacteria have no cultured representative to date.

The project will devote a significant effort to contribute to the development of novel procedures and standards for storing, comparing and annotating metagenomics data. In that respect, the project will be strongly coupled to corresponding efforts in China, and will benefit from a worldwide concerted effort which is monitored by INRA following a consensus workshop held in Paris in October 2005 (Human Intestinal Metagenomics Initiative – Paris Recommendations). The first large set of metagenomics sequence data was released in 2006 (Gill et al 2006) and consisted of 140 000 reads allowing to detect 50 000 genes. The sequencing effort of the present project is far greater with over 2 million reads tentatively leading to identify over 700 000 genes, a sizable fraction of the expected 3 million genes on the basis of 1000 dominant species per human intestinal microbiota.

**The protocol presented below relates to the clinical protocol conducted in France in obese subjects:** Quantification of the relationship between nutritional status, inflammatory and metabolic on one part, and the intestinal microbiota on the other part.
The impact of the nutrition transition on the intestinal microbiota and identification of metagenomic signatures of the nutrition transition will be carried out by other partners of INRA and the group Genoscope in EVRY. Biological samples will be thus collected for further analysis. These include urine and blood samples and preparation of fecal water for carrying out metabolomic analysis and the stools for analysis of proteome of the microbiota (metaproteomics) analyzes. Methodologies are available within the partnership.

# Protection of persons

## Ethical justification of the protocol

It is research for human pathophysiology and of no benefit to the participants. Research is safe for participants if the exclusion criteria are met.

## Ethical and regulatory provisions

The research will be conducted in compliance with French regulations, including provisions relating to biomedical research of the Code of Public Health, Article L 1121-1 and following the laws of Bioethics, the Data Protection Act, Files and Liberties (including the reference methodology MR-001 on biomedical research - Appendix 10) , of the Declaration of Helsinki (Appendix 9) , and of this protocol.
The investigator agrees to conduct the research in accordance with the ethical and regulatory requirements. He/she is aware that all documents and all data relating to the research may be subject to audits and inspections in respect of professional secrecy and may not be resisted without medical confidentiality. The investigator recognizes that the search results are the property of Inserm, the research sponsor.

## Protection Committee (CPP)

Before the implementation of the research, the sponsor will submit the project to the opinion of the Protection Committee (CPP) of the Hospital Dieu and provide the committee with all the necessary information: research protocol, information form and consent (Annex 2), notebook for observations (Annex 3), questionnaires (Annex 3 and 4) and any other relevant documents to be presented to the Committee.

The test will begin when the laboratory CEPRODI KOT has been informed of the favorable unqualified opinion issued by the CPP about the protocol submitted. This review will include the title and protocol number assigned by the sponsor, the documents reviewed and the date of examination and the list of members of the CPP who participated.

The promoter will inform the CPP of all substantive changes to the protocol and of all serious or unexpected adverse events and developments occurring during the research and affecting the safety of persons undergoing the procedures, in accordance with the procedure described in this protocol.

## Insurance and Finance

The CEPRODI-KOT Laboratory, as a sponsor, has subscribed for the entire duration of the test, an insurance liability contract with the company HDI-Gerling Industries, whose address is 111, rue de Longchamp, 75115 Paris, under number 200800080, in accordance with French laws and regulations on biomedical research (insurance certificate relevant to this protocol is annexed to the latter).

The insurance certificate relating to this protocol is contained in Annex 6.

The research funding is provided by the INRA (ANR Research Programme 2008-2011).

## Procedures for obtaining subject consent

Written informed consent of all people who participate in biomedical research must be obtained by the investigator, registered in the order of medical doctors, declared as an investigator with the sponsor before any act can be performed under the research protocol and whatever the act, in accordance with the Public Health Code, Article L1122-1.

Information will be given orally and in writing in the first part of the form of information and consent (Annex 2). The form will be signed at the bottom of each page so that the person suitable for the research confirms receipt of this information. The information is written in plain language understandable to the person. It must contain all the elements that the person must be informed about, in accordance with the Public Health Code, Article L. 1122-1.

The consent of the subject participation in research is collected in writing on the second part of the information and consent form. This second part should be written in clear, understandable language for the person undergoing the research. It must contain all the elements that the person consents. Consent is given by affixing the signature and inscription, from the hand of the person who is willing to take part in the research, first name, last name and date of consent.

In addition, the investigator collects dates and sign the consent form in the position reserved for him and ensures 1) that the form is accurate and no information or date is missing, 2) that an original copy of the document is given to the patient. The investigator maintains a second copy in a safe place and to which access is controlled and 3) a third original copy is placed in a secure location for the attention of the sponsor. The third copy shall not be opened by the sponsor, but retained with the permanent records of the study.

The investigator must ensure that the person is suitable for the research will have time to make a decision freely and be able to read and understand the information form and consent.

The information form and consent is a document that has been approved prior to the implementation of research by the CPP, on the occasion of the review of the protocol.

The participation of people in this protocol shall be registered on the national register of persons undergoing biomedical research, managed by the Ministry of Health.

## Compensation

It is 500 Euros, this amount will be paid at the end of the study, after 12 weeks of participation. If the subject is required to stop the study before completion, no compensation will be paid pro rata.

# ObjectiVEs

**MICRO-Obes** aims to characterize the relationship between intestinal microbiota and the nutritional, inflammatory and metabolic status of the host before and after calorie restriction of 1200 Kcal for 6 weeks followed by a stabilization phase 6 weeks among obese.

**1. A nutritional and metabolic approach will be conducted to assess:** 1) weight loss, 2) reduction of fat mass and adipocyte size, 3) markers of systemic inflammation (CRP-us, IL 6, SAA), 4) plasma lipids, plasma glucose and insulin, 5) the insulin sensitivity, 6) changes in gene and protein expression in adipose tissue (500 mg by suction under local anesthesia with a fine needle) of inflammation factors characteristic of adipocytes and macrophage infiltration.

**2. A metagenomic approach** will also be implemented in order to sequence the dominant genetic repertoire of human intestinal microbiota (stool samples) and identify the functions of the relevant host-microbiota dialogue in capturing energy from food.

# expérimental PLAN

## Type of test

It is to search human pathophysiology. This is a study without direct individual benefit, in obese subjects.

## Category of research :

## Physiopathological study

## Presentation of the experimental design (CF Schéma général : page 2)

A population of 50 obese subjects will be selected after a review of pre inclusion.

After inclusion, subjects follow a low calorie diet of 1200 Kcals for 6 weeks followed by a stabilization phase of 6 weeks. The low calorie diet will be provided and delivered by the laboratory CEPRODI-KOT (attached a list of products offered and a standard menu with products CEPRODI-KOT). Dietary advice will be given at the beginning of each period, patients will be contacted in the middle of each period and a dietician will always contactable by phone or email. The dietician will contact the patient by phone once a week to ensure that there are no difficulties. Data collection will be done at the beginning and end of each period using a food diary kept for one week. The food composition (nutrients) is obtained using a French food composition table.

The stabilization phase of weight will be adjusted depending to the resting energy expenditure (REE) of each patient and will be calculated by the dietician according to the formula of Harris and Benedict :
REE man = 66.473 + (5.003 x height in cm ) + (13.752 x weight in kg) - (6.755 x age)
REE Woman = 655.096 + (1.850 x height in cm ) + (9.563 x weight in kg ) - (4.676 x age)

**Justifications for the selection of a restriction to 1200 kcal for 6 weeks:**

Weight loss by "only" dieting is significant when energy intake is typically significantly lower than energy expenditure. In this study, it will be recommended for subjects to continue their usual level of physical activity. Schemes commonly used in the clinic or in clinical investigation programs are threefold: very low calorie diet (VLCD or very low calorie diet <800 kcal, intermediate (LCD or low calorie diet 800-1500 kcal per day) or very moderate (500 kcal less than usual intakes). Such approaches to the medical management of obesity have been the subject of a recent review in the New England Journal of Medicine (11).

The choice of intermediate diet and duration is based on several arguments: 1) the absence of " risk " associated with a very low calorie diet (VLCD) requiring special monitoring including vitamins and potassium, 2) induction of a slope of rapid weight loss: in previous programs in the team (Project: Effect of two different hypocaloric diets on weight loss and on inflammatory markers) and European programs (**NUGENOB and DIOGENES**), we were able to evaluate this aspect, in agreement with other teams, as weight loss is induced faster in this type of low calorie diet compared with the moderate diet and becomes significant at around 5-6 weeks (eg Figure 1), 3) the feasibility of these programs: these " low calorie diets" were used repeatedly by our team ( Project of Salwa Rizkalla: Effect of two different hypocaloric diets on weight loss and on inflammatory markers) and are well tolerated by the subjects that are regularly monitored by the dietitian in accordance with the recommendations, 4) the observation of a transition of the bacterial flora switch firmicute / bacteroides observed at the limit of significance in a pilot study by our collaborator ANR (Joel Doré).

Therefore we selected a six week diet as the point where even the poor responders monitored under a European project lost about 6% of their initial weight. This is consistent with the work of Gordon Group (12).

**Scientific justification for the 6 weeks of the stabilization phase:**

One of the difficulties of low calorie diets is weight regain after stopping energy restriction and multiple mechanisms involving dialogue inter organ (brain adipose tissue) are set up for a return to the previous weight (11). The predictors of weight maintenance involves the maintenance of a low fat diet, frequent and individual control weight and food intake may be supported by regular advice from a dietician and maintenance of level of physical activity (11).

These techniques will be used to maintain weight loss called the stabilization phase. Maintaining the stabilization phase depends heavily on understanding and adherence to dietary advice. In the previous project (***effets de deux régimes hypocalorique sur la perte du poids et sur les marqueurs de l’inflammation***)), we observed that advice helped a weight maintenance for 8 weeks in 11 of 15 subjects followed. We will continue the one-month follow-up in order to give more opportunities for subjects to maintain their long-term weight gain and use behavioral advice (13).

# évaluation CRITERIA

**Main criteria:**

• Quantification of body weight loss and the loss of total body fat
• Variation in the microbiota Firmicutes / Bacteroidetes
• Quantification of the reduction of systemic inflammation (CRP -us , IL6 , TNF , serum amyloid )
• Analysis of profiles of gene function ( gene regulatory networks analyzed by microarray ) interacting with inflammatory functions in the kinetics of weight loss
• Quantification of macrophage infiltration by gene and protein markers (CD11b CD68, and pro markers (CD40) or anti- inflammatory (CD163, mannose receptor)): in the subcutaneous adipose tissue before and after weight loss and linking with the reduction of systemic inflammation (CRP -us, IL6, TNF, serum amyloid).
• Analysis of interactions between the changes of the intestinal flora, biological variations and adaptations (including inflammatory) adipose tissue.

**Secondary endpoints:**

● Linking changes in the inflammatory profile in adipose tissue and systemic level with clinical and biological changes (including insulin sensitivity).

# Selection of subjects

## Study population:

### Duration of the participation of the people in the research:

The total duration of participation of each subject will be 12 consecutive weeks.

### Description of the participating subjects:

Inclusion Criteria

1 . BMI 27-38 kg/m2
2 . Subjects aged from 25 to 65 years. Postmenopausal for at least 6 months.
3 . Subject has given his consent to participate by writing
4 . Non-diabetic subjects
5 . Fasting blood glucose less than 1.26 g / l

### Exclusion Criteria:

SGOT or SGPT greater than 2.5x the normal value
• High blood glucose:1.26 g / l
• Known heart failure (stage 2 or 3) or confirmed by echocardiography (ejection fraction <60 %) if history of coronary artery disease
• Hepatic impairment known
• Chronic liver disease
• Renal impairment: clearance < 60ml/mn
• Anemia: Hb < 10g/dl
• Participation in another study
• Processing corticosteroids
• Inflammatory Disease
• Any illness making the execution of the protocol or interpretation of results difficult
• Taking antibiotics in the previous 3 months

### Number of people to include:

Effective Calculation: 50 subjects are required to show a change in the ratio of microbiota Firmicutes / Bacteroidetes of at least 40% and a standard deviation of 16 by setting the risk to α β 0.05 and the risk to 0.10 (calculations made after the results of a pilot study with a low calorie diet of 1200 Kcal).

## Recruitment and output test

**Recruitment of participants:**

The subjects will be recruited by public advertisement.

**Exit (drop-out) protocol:**Deliberate decision of a person:
The exit from the study protocol is possible at any time if requested by the participant. More specifically, if participants do not wish to continue the tests, they need only to report to the examiner.

**Decision imposed:**Participants who cannot perform the tasks related to research will be excluded from the protocol.

**Expected Benefits and / or foreseeable risks to persons**No specific individual results are expected from the study. In the exceptional case where, for a given individual, the study would highlight a health risk, relevant data are communicated to the occupational physician who can communicate the most relevant information to the individual case, in the context of respect for persons and medical confidentiality.

**Risks of the diet.**

There are no adverse effects. The products have been marketed for several years. They were used in a previous study conducted in 2007 and no problems were reported.

**Risks related to metabolic investigations.**Biological samples: The possible inconvenience is related to catheter placement and the possibility of bruising. Good permeability of the catheter is provided by the presence of heparin, it is possible that a very small amount of heparin happens in the body without any risk for clotting.

*Aspiration of abdominal fat*: It is performed under local anesthetic, the potential inconvenience can be a hematoma, of modest size that is resolved in a few days.

*Of DXA:* It is safe and provides radiation in the range of 70-140 kV. (less than a chest X-ray). The test takes about ten minutes.

**Constraints of the test.**

The regulation of clinical trials in humans, has forbidden participation in two contradictory experiments simultaneously. If the subject agrees to participate in this study, they should not participate in another trial with diets or drugs during the same period.

# Practical realization of the study

## Detailed description for the acts realised on the participants:

**Nutritional and inflammatory profiles**

We will evaluate lipid profiles and the glycemic contrôle of subjects before and after each periode. At each time point after overnight fast.at 8h00, blood samples will be taken for measurements plasma glucose, insulin, total cholesterol, LDL- and HDL-cholesterol, triglycerides, leptin and adipnectin.Inflammatory markers: CRPus, and IL6 will be also measured by radio immunologic method or by ELISA) and plasma LPS.

Insulin sensitivity will be estimated by a an oral glucose tolerance test after a charge of 75g of glucose .Blood samples will be taken at 30 min , 60 min et 120 min to measure plasma glucose and insulin..

Body fat and fat-free mass distributions were measured using dual-energy X-ray absorptiometry (Hologic APEX, discovery W. (S/N 84030), version 3.0, Bedford, MA

An sample of subcutaneous abdominal adipose tissue will be taken by aspiration with a fine needle under local anaesthesia (1% xylocaine without isoprenalin).

Two supplementary tubes of blood (7 ml) will be taken to measure other new markers that will be identified by the transcriptomic approch

After 7days of the beginning of the dietary program one tube of blood (1 tube of 10 ml) and an adipose tissue biopsy will be taken to evaluate the early kinetic changes of weight loss and the inflammatory markers in adipose tissue.

**Adipose tissue sampling :**

Subcutaneous abdominal adipose tissue samples will be realised by aspiration with a fine needle (300-500 mg) during the inclusion and at the follow up visits. The aspiration of adipose tissue by a needle is a routine test in the department of Nutrition at the Pitié Salpetrière that is realized by an investigator. A fresh aliquot was collected to measure adipocyte diameter, as described previously (14), and another aliquot was stored overnight at 4°C in 4% paraformaldehyde and embedded in paraffin for immunistochemical detection. A third aliquot will be rapidly put in liquid nitrogen then stored at -80°C till the extraction of ARN.

At the end of the study, all the samples will be conserved for future re-evaluation of eventual gene and protein expressions changes.

**Treatment of biological samples**

The serum samples to be assayed for markers of adiposity and inflammatory factors will be analyzed at our Inserm Unit U872 (Eq 7). The serum bank will be stored by the Inserm Unit U872 (Eq 7). The experiments in adipose tissue will be carried out by the Inserm Laboratory U872 (Eq 7) – Research Center in Human Nutrition of Ile de France (Paris 6) who has the necessary equipment for molecular and cellular biology techniques (whole-genome DNA microarray dedicated to inflammation, and real-time quantitative PCR) in collaboration with the functional genomics Inserm network, who develops microarray-specific analysis methods.

1) **Morphology and cellular population**

Adipocyte diameters will be measured for each subject before and after each period.

2) **Gene expression**

The extraction of total ARNs will be done for the freezed tissues using the adapted kit RNasy Mini Kit (QIAGEN, requis, CA) adapté. Due to the small amount of obtained adipose tissue and the difficuklty of rendement, we will use the methode put and validated by the INSER (D Langin), who optimized this method in the European network *MolPage*. On the other hand from total adipose tissue we will identify the candidat genes. Associted to the inflammatory markers, the macrophages (CD 68, CD11b, CD163, mannose receptors, CD40), and the adipokines (leptin, adiponectin), that might be associated to the uinflammatory phenomena.

We will identify also the classes of genetic functions that could be associated to these modifications of the inflammation during body weight loss induced by the hypocaloric diet.

We will study the individual kinetics of gene expression by at day 0, day 7, day 28 and day 42 and after the 6 week stabilization period.

**Urine samples collection:** Samples of urine will be also collected at each time point and conserved at – 20°C.

**Faecal sample collection:** Faecal samples were obtained in the morning at each time point for the determination of metagenomics, metabolomics and metaproteomics. Whole stools were self-collected in sterile boxes and stored at -20°C within 4 h, then sampled as 200-mg aliquots will be moved rapidly to the INRA where it will be stored at -80°C until analysis. Before obtaining the large scale profile of metagenomics, seven groups of the known fecal bacteria will be measured by RT-qPCR. Another global qstudy will be realized by the group of Denis Le Paslier of Genoscope, Evry. We will analyse the reactions between the changes of the intestinal flora, biological changes and the adaptation of the inflammation of adipose tissue by integrating our results to those of the metagebnomic results due to statistic materials and competence concerning the functional genomic (clustering) and multivariant analysis. We have this competence in our unit INSERM U872 by the group of Jean-Daniel Zucker.

## Organization and logistics of the tests

The diet will be provided and delivered weekly by the CEPRODI-KOT laboratory.

Ms. Christine Baudoin in the CRNH-Ile de France contacts the subjects the night before the study visit to remind them of the appointment and the requirement of consuming the standard meal well prepared in advance.

Dietary advice will be given at the beginning of each participation period, patients will be contacted in the middle of each participation period and a dietician will be contactable at all times. Data collection will be done at the beginning and end of each participation period using a food diary kept for a week.

Data will be entered by the investigator on a specific report form. The CRA of the study will verify that the information is complete. Data will be collected. Re-entering the data electronically will allow for the correction of any data entry errors.

## Flow-Chart

- Recruitment period: 8 months.

In case of study withdrawal due to recurring medical problems, the withdrawn subject will not be replaced.

- Total study duration: 24 months.
- The total duration of participation for each subject will be 12 consecutive weeks.

# PRODUCT CONSUMPTION

Each subject will consume a home-prescribed diet.

# MEDICAL DEVICES

This is a dietary intervention.

# COLLECTION OF HUMAN BIOLOGICAL SAMPLES

- Sample description (DNA, cell types, tissues, fluids,…) :

Blood, urine and fecal samples, as well as adipose tissue samples will be obtained at the beginning and end of study participation.

- Description of sample acquisition:

Blood samples will be obtained at days 0, 7, 42 and 84 (fasting). The collection tubes will be placed at 4°C upon sample collection, centrifuged at 4°C, aliquoted and stored at –20°C until analyzed.

Tissue sample collection (abdominal subcutaneous adipose tissue) (300-500 mg) will be done by fine needle aspiration after local anesthesia is applied (Xylocaine). The adipose tissue biopsies will be immediately frozed in liquid nitrogen and stored at -80°C until used for total RNA extraction.

The urine and fecal samples will be immediately frozen at -80 °C and later used for metagenomic, metabolomics and metaproteomic analysis as specified in the MICRO-Obes project.

Samples will be discarded (as usual) after analysis is completed.

# STUDY MONITORING

## Definition of serious adverse events

Definition of a serious adverse event (SAE): An adverse event is qualified as serious if it corresponds to any event that causes the following to a person involved in a clinical trial sponsored by Inserm:

- death,
- an immediate threat to life,
- hospitalization or prolongation of hospitalization,
- disability or a (temporary or permanent clinically significant) significant or prolonged disability,
- a birth anomaly or defect.

Finally, every serious adverse event will be considered as such by the investigator. However, given the nature of this study in which the research is carried during usual visits of occupational medicine, a serious adverse event is extremely unlikely.

A hospitalization already planned as part of the care of the individual will not be considered as a SAE and is not within the scope of this protocol. However, every SAE occurring during said hospitalization, according to the definitions given above, does enter the scope of this protocol.

## Procedure to follow in case of a serious adverse event

The investigator shall promptly notify of a SAE on the day he hears of the SAEs occurred in research:

Using the SAE declaration form that shall be sent by fax or email to the study sponsor.

Then the information will be sent in attention to: Mme Florence Massiera, Laboratoire CEPRODI-KOT, Mission Recherche Clinique, 67 bd de Courcelles, 75008 Paris.

The investigator will also provide any information necessary for the medical assessment of the observation to the sponsor (account of additional tests, pathology, radiology).

The sponsor ask the investigator for additional information.

All documents submitted by the investigator should be de-identified.

The SAE happening after the study should be reported to the sponsor and followed until they are resolved.

The sponsor should report all suspected serious adverse events to the involved CPP and the Direction Générale de la Santé (DGS) without delay and at the latest 7 days after the first day of knowledge of a life-threatening SAE or a SAE leading to death, and within 15 day for other SAE. Any initial statement will be supplemented by a follow-up statement detailing additional relevant information about the SAE in a new period of 8 days after the timing mentioned above.

The sponsor shall send the CPP and DGS an annual safety report taking into account all the safety information available.

The sponsor shall keep detailed records of all adverse events that are reported by the investigators.

In case of occurrence of a new research-related incident that is likely to threat the safety of people involved, the sponsor and the investigator will take appropriate urgent safety measures.

The sponsor shall inform without delay the DGS and CPP of the new incident and, where appropriate, of the measures taken.

## Monitoring of patients in case of occurrence of a serious adverse event

The monitoring of patients that have had a serious adverse event will be provided by the study phycisian.

## New incidents

In case of the occurrence of a new incident related to the research and likely to pose a threat to the safety of the persons involved, the sponsor and investigator shall take appropriate urgent safety measures.

The sponsor shall promptly inform the DGS and CPP of the new incidents and, where appropriate, of the measures taken.

## Independent Monitoring Committee

Research is being conducted during the usual occupational medicine visits, and so there is no need for independent oversight.

# DATA COLLEction and processing

12.1 **Data collection:**

Data in the form of questionnaires will be collected in a report form by the investigators involved in the study. Patient biological sample data will be transcribed using the same system.

**12.2 Data storage prior to electronic data entry:**

The data are stored in locked cabinets on site.

**12.3 Computer data entry:**

Data are digitized as single entries. This will be done under the supervision of Dr. Salwa Rizkalla, scientific officer of the study. Entries are made in an Excel workbook, where each sheet is a different type of parameter. Data will be combined onto a summary sheet and will be exported to the statistical analysis software (SAS). Data safety is ensured by their storage in the 872 Unit server (Team 7), which is secured according to the current rules at Inserm.

**12.4 Description of treatment, verification and validation of the data:**

Computerized data files will be reported to CNIL according to a procedure adapted to this case.

# statistical analysis of the data

**A. Analysis of clinical data and clinical relationships / morphological data and gene expression data.**

Phenotype data will be obtained from the database, anonymously, as usually done. We will analyze the changes in clinical and biological parameters for each individual at baseline and during kinetic monitoring. MANOVA kinetic tests will be used. We will establish correlations between clinical, biological, morphological and gene expression data using logistic regression analysis. In previous years we have acquired the skills to analyze these data (using JMP software, issued by the SAS Institute). For quantitative traits, multivariate analysis will be used (ANOVA, ANCOVA, logistic regression).

1. **Expression studies**

Results from cDNA microarrays will be analyzed by the Inserm Unit U872 (Team 7), where new strategies of oligonucleotide microarray data analysis are being developed. Experimental results will be stored in the SMD database (http://genome-www5.stanford.edu). We will use a certain number of functions developed for R software (steps of filtering, normalization, selection of differentially expressed genes, groups, etc) in order to highlight groups of modified genes. The translation of gene expression data into the corresponding biological mechanisms (commonly called functional analysis or profiling) is considered a major challenge and is an essential step in the elucidation of the biological phenomena underlying the experimental models being studied. Functional annotation will be used to connect sets of genes in the genome with molecular networks that interact within the cell, in order to illustrate the higher order biological information related to metabolic pathways and their regulation. We will search automatic descriptions of enrichment through the databases Gene Ontology (GO) or KEGG (Kyoto Encyclopedia for Genes and Genomes). Our functional analysis will essentially be centered on inflammatory factors and their associated gene networks. Two algorithms, MAGO and FunCluser, have already been designed with this in mind by our team. They automatically link gene transcription microarray data with the genes’ biological roles in order to investigate significant functional changes in the experiment compared to other functions represented on the chip. We will introduce appropriate statistical analysis to take into account multiple testing (False Discovery rate). A computer engineer in the U872 Inserm team will be dedicated to the work of microarray analysis.

# Communication

The results of this study may be presented at scientific conferences or published in medical journals. Publishing projects and findings will be discussed jointly by the principal investigator of this clinical trial coordinator and project coordinator of the ANR (Micro-Obes) and the promoter, their content and their conclusions before final versions of abstracts, texts or posters are fixed.

This restriction also applies to changes that may subsequently be requested by peer reviewers for conferences and journals. The principal investigator, coordinator and promoter also agree not to publish the data in the study without the prior consent of the other parties. In addition, the support of the sponsor must be mentioned.

# SUBSTANTIAL modifications TO THE PROTOCOl

## Procedure of the sponsor on the establishment of an amendment

Any substantial changes to the protocol will become an application for an amendment addressed to the Inserm sponsor, which will be attached to the amended protocol accordingly.

The CPP must be notified of any amendment to the study protocol if the alterations planned change ethical or medico-scientific aspects of the study (endpoint, extension of the period of inclusion, constraints and risks experienced by participants ...).

The amended protocol may also be a request for authorization from the competent authority of the protocol, namely the Directorate General of Health.

# ConfidentialitY

The investigator will ensure that confidentiality regarding each volunteer study participant is guaranteed.

Access to clinical data and sources will be direct in case of audits commissioned by the sponsor or inspections by the competent administrative authorities.

# QUALITY Assurance

The study is conducted in a university hospital experienced in quality control procedures and expertise of stakeholders.

Quality and quality control will be provided by the clinical research team (CRNH - Ile de France by Professor Karine Clément, Dr. Salwa Rizkalla and Christine Baudoin (ARC- APHP).
The quality of the test is governed by a strict compliance with all internal procedures of the institution University Hospital ( Pitié Salpêtrière, Paris), by the application of good clinical practice.
Quality control will be carried out internally every two months.
Quality control will be carried out throughout the study, under the responsibility of the principal investigator and coordinator (Prof. Karine Clement and Dr. Salwa Rizkalla).

This quality control will include:
- The data collected during the study
- The consents of all subjects included
- Materials for the investigation

The relevant information will be accessible to the investigator in case of control.

# REFERENCES

1. **Raben A**, **Holst J**, **Madsen J**, **Astrup A**. Diurnal metabolic profiles after 14 d of an ad libitum high-starch, high-sucrose, or high-fat diet in normal-weight never-obese and postobese women. Am J Clin Nutr 2001;73:177-89.

2. Mutch DM, Wahli W, Williamson G. Nutrigenomics and nutrigenetics: the emerging faces of nutrition

10.1096/fj.05-3911rev. FASEB J. 2005;19:1602-1616.

3. Xu H, Barnes G, Yang Q, et al. Chronic inflammation in fat plays a crucial role in the development of obesity-related insulin resistance. J Clin Invest 2003;112:1821-30.

4. Weisberg S, McCann D, Desai M, Rosenbaum M, Leibel R, Ferrante AJ. Obesity is associated with macrophage accumulation in adipose tissue. J Clin Invest 2003;112:1796-808.

5. Curat C, Miranville A, Sengenes C, et al. From blood monocytes to adipose tissue-resident macrophages: induction of diapedesis by human mature adipocytes. Diabetes 2004;53:1285-92.

6. Laumen H, Skurk T, Hauner H. The HMG-CoA reductase inhibitor rosuvastatin inhibits plasminogen activator inhibitor-1 expression and secretion in human adipocytes. Atherosclerosis 2008;196:565-573.

7. de Luca C, Olefsky JM. Inflammation and insulin resistance. FEBS Letters

Metabolic Disease 2008;582:97-105.

8. Cani P, Delzenne N, .. Gut microflora as a target for energy and metabolic homeostasis. Curr Opin Clin Nutr Metab Care 2007;10:729-34.

9. Cani PD, Bibiloni R, Knauf C, et al. Changes in gut microbiota control metabolic endotoxemia-induced inflammation in high-fat diet-induced obesity and diabetes in mice

10.2337/db07-1403. Diabetes 2008:db07-1403.

10. Cani PD, Delzenne NM, Amar J, Burcelin R. Role of gut microflora in the development of obesity and insulin resistance following high-fat diet feeding. Pathologie Biologie 2008;In Press, Corrected Proof.

11. Eckel RH. Nonsurgical Management of Obesity in Adults

10.1056/NEJMcp0801652. N Engl J Med 2008;358:1941-1950.

12. Backhed F, Manchester JK, Semenkovich CF, Gordon JI. From the Cover: Mechanisms underlying the resistance to diet-induced obesity in germ-free mice

10.1073/pnas.0605374104. Proceedings of the National Academy of Sciences 2007;104:979-984.

13. Astrup A, Rössner S. Lessons from obesity management programmes: greater initial weight loss improves long-term maintenance. Obes Rev 2000;1:17-9.

14. Rizkalla SW, Prifti E, Cotillard A, et al. Differential effects of macronutrient content in 2 energy-restricted diets on cardiovascular risk factors and adipose tissue cell size in moderately obese individuals: a randomized controlled trial. Am J Clin Nutr 2012;95:49-63.
