# Supplementary material for: Dietary Patterns Differently Associate with Inflammation and Gut Microbiota in Overweight and Obese Subjects
Source: PLoS One. 2014 Oct 20;9(10):e109434. doi: 10.1371/journal.pone.0109434 (PMC4203727; doi:10.1371/journal.pone.0109434)
Supplement: Supporting Information S1 — Supporting files. Figure S1, Discriminate canonical analysis (graphical representation of the separation of overweight or obese clusters) with lean subjects projected on the representation. Methods S1,Visualization purposes for Figure 2. Table S1, P values for variance in clinical parameters between lean subjects and all overweight/obese subjects, and between lean subjects and individual clusters. Table S2, P values for variance in food consumption between lean subjects and all overweight/obese subjects, and between lean subjects and individual clusters. Table S3, Percentage of consumers for each food category for lean and overweight and obese subjects. Table S4, Mean daily nutrient intakes for lean, overweight/obese subjects and dietary clusters. Table S5, P values for variance in nutrient intakes between lean subjects and all overweight/obese subjects, and between lean subjects and individual clusters. Table S6, Differences in gut microbiota (qPCR) in lean, overweight/obese subjects and in the 3 dietary clusters. Table S7, P values for variance in gut microbiota (qPCR) between lean subjects and all overweight/obese subjects, and between lean subjects and individual clusters. Table S8, Correlations between the 7 gut bacterial groups measured by the qPCR and the intake of food groups without stratification to clusters. Heatmap of correlations between qPCR data and food categories. Table S9, Differences in gene richness group (LGC/HGC subjects) in the 3 dietary clusters. (DOC) [file pone.0109434.s003.doc]

**SUPPORTING INFORMATION S1:**

**Figure S1: Discriminate canonical analysis (graphical representation of the separation of overweight or obese clusters) with lean subjects projected on the representation.**


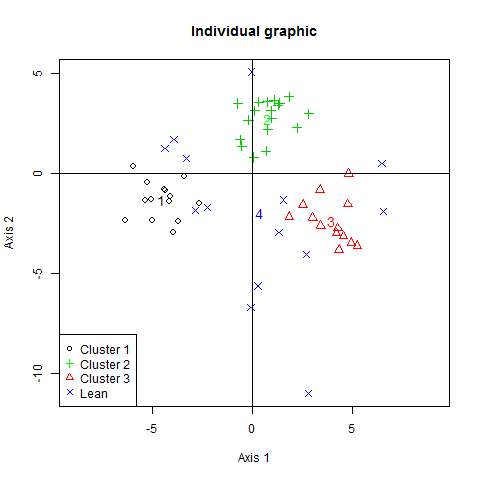


Cluster 1: black diamond; Cluster 2: green crosses; Cluster 3: red triangles, Lean subjects blue crosses.

**Table S1: *P* values for variance in clinical parameters between lean subjects and all overweight/obese subjects, and between lean subjects and individual clusters.**

|  | ***P* vs Ob_all***  ***N=45*** | ***P* vs cluster 1***  ***N=14*** | ***P* vs cluster 2***  ***N=18*** | ***P* vs cluster3***  ***N=13*** |
| --- | --- | --- | --- | --- |
| **Adiposity markers** |  |  |  |  |
| Body weight (kg) | <0.001 | <0.001 | <0.001 | <0.001 |
| BMI (kg/m^2^) | <0.001 | <0.001 | <0.001 | <0.001 |
| Total Fat mass% | <0.001 | <0.001 | <0.001 | <0.001 |
| Waist circumference (cm) | <0.001 | <0.001 | <0.001 | <0.001 |
| Adipocyte diameter (µm) | <0.001 | <0.001 | <0.001 | <0.001 |
| Leptin (ng/ml) | <0.001 | <0.001 | <0.001 | <0.001 |
| **Plasma glucose homeostasis and insulin sensitivity** |  |  |  |  |
| Fasting glycaemia (mmol/l) | <0.001 | <0.001 | <0.001 | <0.001 |
| Fasting insulinaemia (µU/ml) | 0.03 | 0.1 | 0.02 | 0.38 |
| HOMA-IR | 0.01 | 0.07 | 0.01 | 0.26 |
| Adiponectin (µg/ml) | 0.01 | 0.01 | 0.006 | 0.05 |
| **Plasma lipid homeostasis** |  |  |  |  |
| Total Cholesterol (mmol/l) | <0.001 | 0.002 | <0.001 | 0.03 |
| HDL Cholesterol (mmol/l) | 0.19 | 0.12 | 0.5 | 0.2 |
| LDL cholesterol (mmol/l) | <0.001 | <0.001 | 0.002 | 0.04 |
| Triglycerides (mmol/l) | <0.001 | <0.001 | <0.001 | <0.001 |
| Fasting FFA (mmol/l) | 0.01 | 0.03 | 0.04 | 0.1 |
| **Inflammatory markers** |  |  |  |  |
| hsCRP (mg/l) | 0.002 | 0.02 | 0.01 | 0.03 |
| IL6 (pg/ml) | 0.1 | 0.03 | 0.7 | 0.2 |
| LPS (EU/ml) | 0.1 | 0.1 | 0.5 | 0.04 |
| sCD14 (ng/ml) | 0.9 | 0.1 | 0.9 | 0.4 |
| **Adipose tissue macrophages** |  |  |  |  |
| HAM56 | 0.07 | 0.47 | 0.07 | 0.08 |
| HAM56% | 0.01 | 0.2 | 0.02 | 0.01 |
| CD163 | 0.4 | 0.04 | 0.4 | 0.4 |
| CD163% | 0.9 | 0.2 | 0.74 | 0.2 |
| **Chemokines** |  |  |  |  |
| MCP-1 (pg/ml) | 0.04 | 0.7 | 0.08 | 0.008 |
| VEGF (pg/ml) | 0.8 | 0.6 | 0.7 | 0.4 |
| Eotaxin (pg/ml) | 0.1 | 0.08 | 0.4 | 0.3 |
| IP10 (µg/ml) | 0.001 | <0.001 | 0.09 | 0.01 |
| MIP-1b (pg/ml) | 0.6 | 0.5 | 0.6 | 0.8 |

***** Wilcoxon rank sum test stands for variance between lean subjects and all overweight and obese subjects, and between lean subjects and the individual clusters.

**Table S2: *P* values for variance in food consumption between lean subjects and all overweight/**obese subjects, and between lean subjects and individual clusters.

|  | **Lean vs. All overweight and obese** | **Lean vs. Cluster 1** | **Lean vs. Cluster 2** | **Lean vs. Cluster 3** |
| --- | --- | --- | --- | --- |
| **Food category** | ***P**** | ***P**** | ***P**** | ***P**** |
| Bread and bread products | 0.936 | 0.782 | 0.834 | 0.423 |
| Cereals e.g. rice, pasta | 0.103 | 0.182 | 0.246 | 0.138 |
| Pulses e.g. lentils | 0.401 | 0.365 | 0.508 | 0.594 |
| Potatoes including chips | 0.452 | 0.036 | 0.344 | 0.341 |
| Breakfast cereals | 0.288 | 0.043 | 0.735 | 1.000 |
| Milk | 0.296 | 0.139 | 0.937 | 0.258 |
| Yogurt****** | 0.153 | 0.003 | 0.790 | 0.752 |
| Cheese | 0.803 | 0.945 | 0.481 | 0.903 |
| White meat e.g. chicken | 0.020 | 0.434 | 0.044 | 0.003 |
| Red meat e.g. beef, lamb | 0.742 | 0.489 | 0.985 | 0.845 |
| Delicatessen meats e.g. ham | 0.574 | 0.945 | 0.177 | 0.922 |
| Fish and fish products | 0.237 | 0.088 | 0.554 | 0.543 |
| Fruit | 0.859 | 0.006 | 0.747 | 0.005 |
| Vegetables | 0.084 | 0.629 | 0.052 | 0.007 |
| Fats and oils | 0.373 | 0.102 | 0.238 | 0.395 |
| Eggs and egg dishes | 0.102 | 0.734 | 0.014 | 0.350 |
| Sweets, confectionary and table sugar | 0.218 | ***0.001*** | 0.864 | 0.610 |
| Pastries and sweet biscuits | 0.475 | 0.290 | 0.278 | 0.543 |
| Soups | 0.107 | 0.065 | 0.025 | 0.788 |
| Savoury snacks, pies and pizzas | 0.375 | 0.865 | 0.293 | 0.210 |
| Condiments and sauces | 0.004 | 0.005 | 0.006 | 0.116 |
| Nuts and seeds | 0.419 | 0.578 | 0.241 | 1.000 |
| Water (all types) | ***0.001*** | 0.549 | ***0.000*** | 0.005 |
| Drinks without sugar without alcohol e.g. tea, coffee | ***0.001*** | 0.006 | ***0.002*** | 0.013 |
| Drinks with sugar without alcohol e.g. soda, fruit juice | 0.025 | ***0.001*** | 0.034 | 0.940 |
| Drinks with alcohol e.g. wine, beer | 0.653 | 0.735 | 0.388 | 0.663 |

*****Kruskal-Wallis rank sum test with Bonferroni correction, *P* value significant at ≤0.002 is shown in bold italics. Wilcoxon rank sum test stands for variance between lean subjects and all overweight and obese subjects, and between lean subjects and the individual clusters.

******This group contains other fermented dairy products, e.g. white cheese.

**Table S3: Percentage of consumers for each food category for lean and overweight and obese subjects.**

|  | **Lean (n=14)** | **All overweight and obese (n=45)** | **Overweight and obese dietary clusters** | | |
| --- | --- | --- | --- | --- | --- |
|  |  |  | **Cluster 1 (n=14)** | **Cluster 2 (n=18)** | **Cluster 3 (n=13)** |
| **Food category** | % | % | % | % | % |
| Bread and bread products | 93 | 96 | 100 | 94 | 92 |
| Cereals e.g. rice, pasta | 100 | 93 | 86 | 94 | 100 |
| Pulses e.g. lentils | 7 | 18 | 21 | 17 | 15 |
| Potatoes including chips | 71 | 84 | 100 | 67 | 92 |
| Breakfast cereals | 7 | 20 | 43 | 11 | 8 |
| Milk | 57 | 69 | 71 | 56 | 85 |
| Yogurt***** | 86 | 89 | 71 | 100 | 92 |
| Cheese | 86 | 87 | 93 | 83 | 85 |
| White meat e.g. chicken | 43 | 93 | 79 | 100 | 100 |
| Red meat e.g. beef, lamb | 93 | 98 | 100 | 94 | 100 |
| Delicatessen meats e.g. ham | 71 | 96 | 93 | 100 | 92 |
| Fish and fish products | 79 | 78 | 57 | 83 | 92 |
| Fruit | 100 | 93 | 79 | 100 | 100 |
| Vegetables | 93 | 100 | 100 | 100 | 100 |
| Fats and oils | 100 | 100 | 100 | 100 | 100 |
| Eggs and egg dishes | 29 | 67 | 50 | 83 | 62 |
| Sweets, confectionary and table sugar | 93 | 82 | 100 | 67 | 85 |
| Pastries and sweet biscuits | 86 | 84 | 86 | 89 | 77 |
| Soups | 57 | 53 | 43 | 33 | 92 |
| Savoury snacks, pies and pizzas | 50 | 47 | 57 | 44 | 38 |
| Condiments and sauces | 21 | 80 | 86 | 83 | 69 |
| Nuts and seeds | 7 | 16 | 14 | 22 | 8 |
| Water (all types) | 86 | 96 | 86 | 100 | 100 |
| Drinks without sugar without alcohol e.g. tea, coffee | 100 | 87 | 64 | 100 | 92 |
| Drinks with sugar without alcohol e.g. soda, fruit juice | 57 | 82 | 100 | 83 | 62 |
| Drinks with alcohol e.g. wine, beer | 36 | 53 | 43 | 67 | 46 |

***** This group contains other fermented dairy products, e.g. fromage blanc.

**Table S4: Mean daily nutrient intakes for lean, overweight/obese subjects and dietary clusters**

|  | **Lean (n=14)** | **All overweight and obese (n=45)** | **Overweight and obese dietary clusters** | | |  |
| --- | --- | --- | --- | --- | --- | --- |
| **Nutrient (unit)** |  |  | **Cluster 1 (n=14)** | **Cluster 2 (n=18)** | **Cluster 3 (n=13)** | ***P**** |
| Energy (kcal) | 1616.65 ± 110.45 | 1668.38 ± 54.20 | 1763.96 ± 122.08 | 1611.14 ± 74.83 | 1644.72 ± 87.58 | 0.507 |
| Water (g) | 1732.57 ± 121.36 | 1938.92 ± 79.29 | 1671.27 ± 105.85 | 2063.71 ± 147.11 | 2054.37 ± 122.22 | 0.036 |
| Protein (g) | 75.32 ± 6.68 | 77.33 ± 3.07 | 70.35 ± 4.49 | 81.07 ± 5.93 | 79.68 ± 4.51 | 0.332 |
| Protein (% energy) | 18.68 ± 1.21 | 19.11 ± 0.71 | 16.64 ± 0.91 | 20.62 ± 1.38 | 19.67 ± 0.93 | 0.034 |
| Carbohydrate (g) | 176.22 ± 15.03 | 177.62 ± 7.45 | 196.05 ± 16.96 | 160.83 ± 9.57 | 181.03 ± 11.15 | 0.176 |
| Carbohydrate (% energy) | 43.53 ± 2.39 | 42.51 ± 1.01 | 43.93 ± 1.68 | 39.96 ± 1.69 | 44.53 ± 1.63 | 0.142 |
| Total Fat (g) | 64.31 ± 5.77 | 69.57 ± 2.79 | 75.97 ± 5.70 | 68.32 ± 4.06 | 64.40 ± 4.73 | 0.356 |
| Total fat (% energy) | 35.55 ± 1.55 | 37.10 ± 0.75 | 38.54 ± 1.23 | 37.76 ± 1.07 | 34.63 ± 1.52 | 0.116 |
| Saturated fat (g) | 23.41 ± 1.53 | 23.97 ± 1.19 | 26.32 ± 2.67 | 23.24 ± 1.63 | 22.44 ± 1.92 | 0.535 |
| Monounsaturated fat (g) | 21.48 ± 2.28 | 22.92 ± 1.05 | 23.12 ± 1.92 | 21.72 ± 1.20 | 24.38 ± 2.54 | 0.533 |
| Polyunsaturated fat (g) | 7.55 ± 1.02 | 9.09 ± 0.68 | 7.50 ± 1.00 | 10.94 ± 1.16 | 8.24 ± 1.19 | 0.074 |
| Alcohol (g) | 3.60 ± 1.46 | 2.80 ± 0.58 | 1.17 ± 0.54 | 3.56 ± 0.89 | 3.50 ± 1.44 | 0.207 |
| Fibre (g) | 14.13 ± 1.46 | 14.53 ± 0.76 | 11.32 ± 0.67 | 14.25 ± 1.19 | 18.38 ± 1.36 | ***0.001***^†^ |
| Vitamin A (µg) | 299.25 ± 45.96 | 400.04 ± 66.56 | 264.88 ± 33.39 | 537.33 ± 133.63 | 355.52 ± 126.1 | 0.131 |
| Betacarotene (µg) | 3540.47 ± 840.20 | 3753.68 ± 439.01 | 3397.11 ± 833.20 | 3797.47 ± 722.64 | 4077.06 ± 769.14 | 0.743 |
| Vitamin B1 (mg) | 0.84 ± 0.09 | 1.05 ± 0.05 | 1.00 ± 0.07 | 1.13 ± 0.10 | 1.01 ± 0.09 | 0.784 |
| Vitamin B2 (mg) | 1.20 ± 0.10 | 1.33 ± 0.07 | 1.25 ± 0.13 | 1.38 ± 0.13 | 1.34 ± 0.10 | 0.485 |
| Vitamin B3 (mg) | 14.27 ± 1.27 | 14.54 ± 0.64 | 13.12 ± 1.15 | 14.94 ± 1.05 | 15.52 ± 1.07 | 0.359 |
| Vitamin B5 (mg) | 3.56 ± 0.33 | 3.71 ± 0.20 | 2.93 ± 0.18 | 3.85 ± 0.37 | 4.36 ± 0.29 | 0.009 |
| Vitamin B6 (mg) | 1.38 ± 0.11 | 1.38 ± 0.07 | 1.16 ± 0.09 | 1.37 ± 0.11 | 1.62 ± 0.12 | 0.016 |
| Vitamin B9 (µg) | 219.77 ± 25.88 | 243.11 ± 12.36 | 195.28 ± 16.76 | 259.69 ± 20.91 | 271.66 ± 21.21 | 0.024 |
| Vitamin B12 (µg) | 4.16 ± 0.75 | 4.09 ± 0.41 | 3.10 ± 0.45 | 4.83 ± 0.81 | 4.12 ± 0.68 | 0.270 |
| Vitamin C (mg) | 76.79 ± 10.25 | 93.37 ± 6.56 | 60.31 ± 7.91 | 99.56 ± 9.26 | 120.39 ± 11.93 | ***0.001***^†‡^ |
| Vitamin D (µg) | 2.69 ± 0.62 | 1.72 ± 0.19 | 1.44 ± 0.22 | 2.12 ± 0.34 | 1.45 ± 0.38 | 0.197 |
| Vitamin E (mg) | 7,91 ± 1.01 | 8.46 ± 0.58 | 6.06 ± 0.56 | 10.15 ± 1.04 | 8.71 ± 0.94 | 0.002 |
| Sodium (mg) | 2201.67 ± 211.21 | 2112.32 ± 91.31 | 1976.28 ± 128.39 | 2083.94 ± 149.63 | 2298.13 ± 195.06 | 0.492 |
| Potassium (mg) | 2524.69 ± 182.40 | 2548.17 ± 93.40 | 2273.44 ± 112.44 | 2472.07 ± 140.25 | 2949.41 ± 191.16 | 0.021 |
| Magnesium (mg) | 234.41 ± 15.03 | 221.97 ± 7.65 | 202.62 ± 11.34 | 219.73 ± 11.07 | 245.91 ± 16.42 | 0.176 |
| Phosphorus (mg) | 1000.55 ± 72.36 | 1033.55 ± 40.80 | 961.43 ± 59.49 | 1054.78 ± 73.05 | 1081.84 ± 76.29 | 0.561 |
| Calcium (mg) | 715.21 ± 60.57 | 743.41 ± 42.22 | 742.02 ± 67.92 | 742.29 ± 70.65 | 746.44 ± 86.28 | 0.997 |
| Iron (mg) | 9.52 ± 0.99 | 9.78 ± 0.43 | 8.64 ± 0.67 | 9.83 ± 0.53 | 10.93 ± 0.99 | 0.123 |

Data are presented as means ± SEM.

*****Kruskal-Wallis rank sum test with Bonferroni correction, *P* value significant at ≤0.002 is shown in bold italics. Wilcoxon rank sum test stands for variance between overweight/obese individual clusters. ^†^significant difference between Clusters 1 and 3.^‡^ significant difference between Clusters 1 and 2; ^§^significant difference between Clusters 2 and 3. *P* values testing variance between lean subjects and all overweight and obese subjects and between lean subjects and the individual clusters are shown in **Table S5.**

**Table S5: *P* values for variance in nutrient intakes between lean subjects and all overweight/**obese subjects, and between lean subjects and individual clusters.

|  | **Lean vs. All overweight and obese** | **Lean vs. Cluster 1** | **Lean vs. Cluster 2** | **Lean vs. Cluster 3** |
| --- | --- | --- | --- | --- |
| **Nutrient (unit)** | ***P**** | ***P**** | ***P**** | ***P**** |
| Energy (kcal) | 0.423 | 0.306 | 0.779 | 0.519 |
| Water (g) | 0.165 | 0.701 | 0.071 | 0.061 |
| Protein (g) | 0.789 | 0.667 | 0.561 | 0.685 |
| Protein (% energy) | 0.643 | 0.246 | 0.283 | 0.280 |
| Carbohydrate (g) | 0.972 | 0.376 | 0.377 | 0.943 |
| Carbohydrate (% energy) | 0.631 | 0.910 | 0.193 | 0.830 |
| Total Fat (g) | 0.187 | 0.069 | 0.442 | 0.550 |
| Total fat (% energy) | 0.487 | 0.210 | 0.419 | 0.616 |
| Saturated fat (g) | 0.929 | 0.427 | 0.837 | 0.756 |
| Monounsaturated fat (g) | 0.373 | 0.401 | 0.750 | 0.302 |
| Polyunsaturated fat (g) | 0.412 | 0.839 | 0.059 | 0.981 |
| Alcohol (g) | 0.850 | 0.593 | 0.487 | 0.894 |
| Fibre (g) | 0.845 | 0.150 | 0.896 | 0.061 |
| Vitamin A (µg) | 0.682 | 0.982 | 0.116 | 0.350 |
| Betacarotene (µg) | 0.817 | 0.874 | 0.866 | 0.583 |
| Vitamin B1 (mg) | 0.094 | 0.227 | 0.084 | 0.325 |
| Vitamin B2 (mg) | 0.383 | 0.635 | 0.464 | 0.402 |
| Vitamin B3 (mg) | 0.972 | 0.482 | 0.837 | 0.583 |
| Vitamin B5 (mg) | 0.557 | 0.352 | 0.561 | 0.068 |
| Vitamin B6 (mg) | 0.943 | 0.227 | 1.000 | 0.141 |
| Vitamin B9 (µg) | 0.233 | 0.667 | 0.125 | 0.085 |
| Vitamin B12 (µg) | 0.915 | 0.306 | 0.639 | 0.867 |
| Vitamin C (mg) | 0.226 | 0.114 | 0.071 | 0.007 |
| Vitamin D (µg) | 0.336 | 0.346 | 0.864 | 0.182 |
| Vitamin E (mg) | 0.762 | 0.210 | 0.156 | 0.756 |
| Sodium (mg) | 0.695 | 0.427 | 0.722 | 0.793 |
| Potassium (mg) | 0.845 | 0.246 | 0.985 | 0.085 |
| Magnesium (mg) | 0.498 | 0.210 | 0.536 | 0.756 |
| Phosphorus (mg) | 0.605 | 0.982 | 0.667 | 0.458 |
| Calcium (mg) | 0.708 | 0.541 | 0.837 | 0.981 |
| Iron (mg) | 0.631 | 0.511 | 0.561 | 0.220 |

*****Kruskal-Wallis rank sum test with Bonferroni correction, *P* value significant at ≤0.002 is shown in bold italics. Wilcoxon rank sum test stands for variance between lean subjects and all overweight and obese subjects, and between lean subjects and the individual clusters.

******This group contains other fermented dairy products, e.g. fromage blanc.

**Table S6: Differences in gut microbiota (qPCR) in lean, overweight/obese subjects and in the 3 dietary clusters**

|  | **Lean subjects** | **All subjects**  **(n=45)** | **Dietary pattern** | | |  | |
| --- | --- | --- | --- | --- | --- | --- | --- |
|  |  |  | **Cluster 1 (N=14)** | **Cluster 2 (N=18)** | **Cluster 3 (N=13)** | ***Kruskal P* *** | ***Trend P***** |
| **Gut microbiota (qPCR)** |  |  |  |  |  |  |  |
| All Bacteria | 11.21±0.04 | 11.35±0.05 | 11.29±0.06 | 11.37±0.01 | 11.4±0.08 | 0.61 | 0.53 |
| *Clostridium leptum* group | -0.4±0.06 | -0.84±0.11 | -1.07±0.33 | -0.73±0.09 | -0.87±0.12 | 0.81 | 0.71 |
| Clostridium coccoides group | -1.97±0.08 | -2.57±0.08 | -2.69±0.22 | -2.52±0.13 | -2.51±0.11 | 0.92 | 0.95 |
| *Lactobacillus/Leuconostoc/Pediococcus* group | -4.27±0.2 | -4.05±0.1 | -3.76±0.26 | -4.25±0.09 | -4.2±0.13 | 0.47 | 0.39 |
| *Faecalibacteriumprausnitzii* species | -1.13±0.2 | *-*1.42±0.09 | -1.62±0.29 | -1.39±0.08 | -1.31±0.07 | 0.93 | 0.71 |
| *Bifidobacterium* genus | -2.75±0.23 | -2.62±0.12 | -2.43±0.21 | -2.71±0.21 | -2.86±0.22 | 0.84 | 0.76 |
| *Bacteroides/Prevotella* group | -0.69±0.07 | -1.47±0.05 | -1.52±0.11 | -1.44±0.07 | -1.47±0.12 | 0.48 | 0.34 |
| *E. coli* species | -3.6±0.14 | -3.87±0.15 | -3.99±0.26 | -3.98±0.23 | -3.69±0.37 | 0.78 | 0.50 |

Data are means ± SEM; n= 45 subjects; HAM56%=the cell number marked by antibody anti-HAM56/the adipocytes number; CD163%=the cell number marked by antibody anti-CD163/the adipocytes number; *stratified Kruskal-Wallis rank sum test stands for variance among 3 groups at each time point. ^**^tests for trend staratified by age; *P* value ≤0.05 was marked in bold and italic. 0.05<*P* value<0.15 is shown in italics. Stratified post-hoc Nemenyi tests stands for variance between each set of 2 clusters: ^†^significant difference between Clusters 1 and 3. Results of gut microbiota detected by qPCR method were expressed as mean of the log10 value ± SEM of normalized data, calculated as the log number of targeted bacteria minus the log number of all bacteria.

**Table S7: *P* values for variance in gut microbiota (qPCR) between lean subjects and all overweight/obese subjects, and between lean subjects and individual clusters.**

|  | ***P* vs Ob_all***  ***N=45*** | ***P* vs cluster 1***  ***N=14*** | ***P* vs cluster 2***  ***N=18*** | ***P* vs cluster3***  ***N=13*** |
| --- | --- | --- | --- | --- |
| All Bacteria | 0.07 | 0.4 | 0.09 | 0.09 |
| *Clostridia leptum* group | 0.005 | 0.07 | 0.01 | 0.003 |
| *Clostridia coccoides* group | <0.001 | <0.001 | 0.002 | 0.001 |
| *Lactobacillus/Leucoconostoc/Pediococcus* group | 0.1 | 0.1 | 0.2 | 0.3 |
| *Faecalibacteria prausnitzii* species | 0.08 | 0.1 | 0.1 | 0.2 |
| *Bifidobacterium* genus | 0.8 | 0.4 | 0.9 | 0.5 |
| *Bacteroides/Prevotella* group | <0.001 | <0.001 | <0.001 | <0.001 |
| *E. coli* species | 0.46 | 0.25 | 0.4 | 0.9 |

***** Wilcoxon rank sum test stands for variance between lean subjects and all overweight and obese subjects, and between lean subjects and the individual clusters.

**Table S8:** Correlations between the 7 gut bacterial groups measured by the qPCR and the intake of food groups without stratification to clusters. Heatmap of correlations between qPCR data and food categories.

Values are p-values adjusted by Benjamini-Hochberg correction for each qPCR group. The color correspond to the rho of the correlation: When green, it indicates a negative correlation and when it is red it is a positive one.

**Table S9: Differences in gene richness group (LGC/HGC subjects) in the 3 dietary clusters**

| Gene count groups |  | Dietary pattern | | |
| --- | --- | --- | --- | --- |
|  |  | Cluster 1 | Cluster 2 | Cluster 3 |
| HGC  LGC |  | 0.43 | 0.5 | 0.83 |
|  |  | 0.57 | 0.5 | 0.17 |

Data are the frequencies of each gene count group among the dietary clusters.

**Supplementary methods S1:**

For visualization purposes, in Figure 2, mean and SEM values in each food cluster were adjusted for age groups in the same way as death rates have previously been (Age standardization of death rates: implementation of the year 2000 standard, Anderson RN, Rosenberg HM, Natl Vital Stat Rep. 1998 Oct 7;47(3):1-16, 20).

Let’s X be the clinical variable of interest, A and C be respectively the age and food cluster groups taking values in {a_1_, a_2_, a_3_} and {c_1_, c_2_, c_3_}. Let’s also define P_A_ as the age group distribution in the global population of 45 subjects. Then, when adjusting by age group, means were computed as

$E_{P_{A}}\left(X\left|C=c_{i}\right.\right)=\sum_{i=1}^{3} P(A=a_{i})\times E\left(X\left|C=c_{i},A=a_{i}\right.\right)$,

and variances were computed as

$\begin{matrix} V_{P_{A}}\left(X\left|C=c_{i}\right.\right)=E_{P_{A}}\left(X^{2}\left|C=c_{i}\right.,A\right)-\left(E_{P_{A}}\left(X\left|C=c_{i},A\right.\right)\right)^{2} \\ =\sum_{i=1}^{3} P(A=a_{i})\times E\left(X^{2}\left|C=c_{i},A=a_{i}\right.\right)-\left(\sum_{i=1}^{3} P(A=a_{i})\times E\left(X\left|C=c_{i},A=a_{i}\right.\right)\right)^{2} \\ =\sum_{i=1}^{3} P(A=a_{i})\times\left(E\left(X\left|C=c_{i},A=a_{i}\right.\right)^{2}+V\left(X\left|C=c_{i},A=a_{i}\right.\right)\right)-\left(\sum_{i=1}^{3} P(A=a_{i})\times E\left(X\left|C=c_{i},A=a_{i}\right.\right)\right)^{2} \end{matrix}$SEM values were then obtained by $sem_{c_{i}}=\sqrt{\frac{V_{c_{i}}}{n_{c_{i}}}},$where n_ci_ is the number of patients in group c_i_.
